# Molecular Sensing of Chiral Carboxylic Acid Enantiomers Using CD Inductions in the Visible Light Region

**DOI:** 10.1021/acs.joc.4c02055

**Published:** 2025-01-02

**Authors:** Jeffrey S. S. K. Formen, Eryn Nelson, Christian Wolf

**Affiliations:** Chemistry Department, Georgetown University, Washington, D.C. 20057, United States

## Abstract

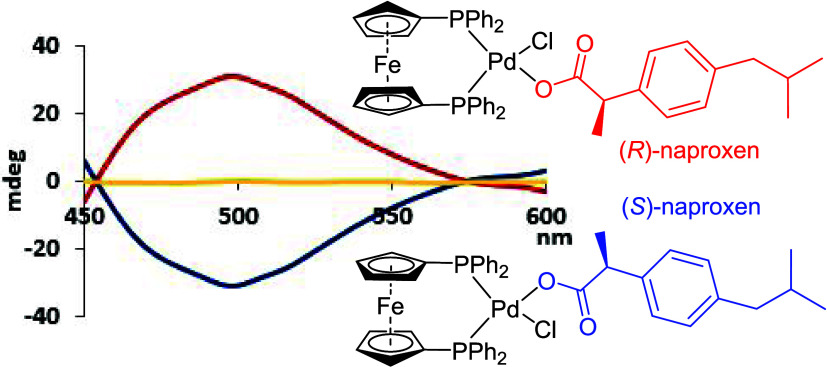

The reaction between a chiral carboxylic acid molecule and 1,1′-bis(diphenylphosphino)ferrocenepalladium dichloride in the presence of a mild base generates a chiroptically active metal complex displaying strong circular dichroism (CD) signals in the visible light region, a highly sought-after goal in the optical sensing realm. The molecular recognition process is complete within a few minutes and can be used for fast chiroptical determination of the enantiomeric composition and concentration of carboxylic acid samples. This method is operationally simple and broadly applicable to a large variety of structures including important drugs, natural products, amino acids, and hydroxy acids. All components needed are commercially available, and this optical sensing assay can be readily adapted by any laboratory interested in chirality analysis and high-throughput experimentation.

## Introduction

Chiral carboxylic acids exist widely in nature, where they play fundamental roles in a fascinating blend of chemical processes and complex biological systems. They are also in high demand as versatile synthetic building blocks or invaluable precursors of multifunctional natural products and are frequently encountered moieties in important biologically active compounds, including ibuprofen, naproxen, tiagabine, abietic acid, isosteviol, and dihydroartemisinic acid. The general significance, structural variety, and broad application scope of chiral carboxylic acids are truly remarkable and continue to attract widespread attention across the chemical and health sciences. As the pharmacological activity or utility is often closely connected with the chirality of carboxylic acids, many laboratories regularly face the task of determining or monitoring their enantiomeric composition, preferably by processing a large number of samples in an automated high-throughput fashion. Chiral chromatography and NMR spectroscopic methods with chiral derivatizing reagents or solvating agents are the current gold standard but often cause a bottleneck in compound development projects because they are intrinsically serial techniques and cannot match high-throughput screening (HTS) expectations. Accordingly, methods based on mass spectrometry,^1^ UV,^2^ fluorescence,^3−5^ gas-phase rotational resonance,^6^ IR,^7^ electronic circular dichroism (ECD),^8−11^ fluorescence-detected CD spectroscopy,^12^ and biochemical assays^13^ have been introduced. In recent years, several groups have demonstrated that chiroptical sensing methods are particularly useful.^14−16^ A variety of chromophoric sensors that undergo stoichiometric interactions with a chiral acid molecule, e.g., formation of a supramolecular assembly,^17,18^ hydrogen bonding,^19−21^ or salt bridges,^22^ to generate a strong circular dichroism (CD) signal for accurate determination of the sample enantiomeric ratio (*er*) have been introduced. The use of achiral sensors is particularly attractive because they avoid the formation of diastereomers and take full advantage of the inherently enantiodifferentiating nature of CD spectroscopy. In some cases, concomitant induction of nonenantioselective UV or fluorescence signals allows quantification of the total amount of both enantiomers in addition to the *er* value.

Unsurprisingly, the large majority of chiroptical assays that have been developed to date target α-amino acids^23−38^ and α-hydroxy acids,^39−48^ which are privileged bidentate structures favoring effective CD signal inductions. Despite the introduction of sensing systems that can differentiate between enantiomers of monofunctional analytes,^17−22,49−53^ a widely applicable optical method that is complete within a few minutes, based on a simple mixing protocol with readily available assay components, and allows combined concentration and *er* determination solely from far red-shifted CD inductions with maxima appearing in the visible light region has not been reported. To this end, an achiral probe generating a strong CD maximum beyond 450 nm upon binding of a chiral carboxylic acid would be most useful because this simplifies the analytical task as diastereomer formation can be ruled out, decreases the risk of possible interferences from chiral impurities that may have intrinsic CD signatures at shorter wavelengths, and is more amenable to the use in automated CD plate readers that have technical issues with measurements around 400 nm.^54^

## Results and Discussion

**Figure 1:**
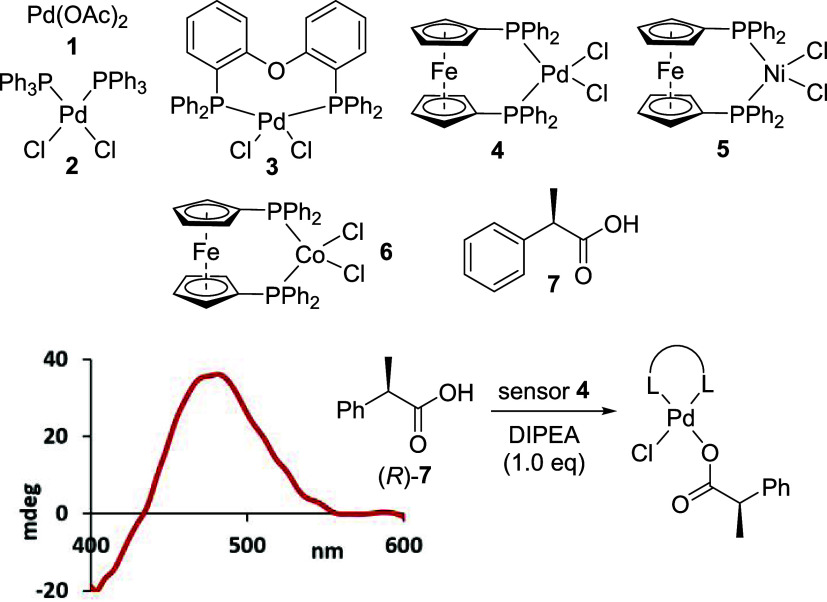
Structures of the sensors investigated and CD induction obtained by coordination of (*R*)-2-phenylpropanoic acid to Pd complex **4**.

During our search for a molecular sensor that would fulfill all of these expectations, we considered the potential of metal coordination chemistry using the sensors **1**–**6** and 2-phenylpropanoic acid, **7**, as test analytes. Indeed, we found that the formation of a palladium(II) complex by mixing (*R*)-**7** and Pd(OAc)_2_ in the presence of a tertiary amine generates a respectable CD maximum at around 320 nm (Supporting Information). With this encouraging proof-of-concept in hand, we sought to include chromophoric phosphine ligands to favor stoichiometric carboxylate binding, which we expected would facilitate quantitative sensing applications, and concomitant induction of a strong CD signal at significantly longer wavelengths. While we had limited success with (PPh_3_)_2_PdCl_2_, we observed CD inductions above 400 nm with [bis(2-(diphenylphosphino)phenyl)ether]PdCl_2_, **3**, and its 1,1′-bis(diphenylphosphino)ferrocene analogue, **4**. The strong, red-shifted chiroptical response obtained with **4** is particularly impressive and a rare example of ligand-enhanced CD induction occurring in the visible light region. We were delighted to record an induced CD (ICD) maximum of approximately 40 °C, appearing around 475 nm at 2.65 mM in tetrahydrofuran (Figure 1). Moreover, palladium complex **4** is commercially available and soluble in common organic solvents. These noteworthy features underscore the practicality of this chiroptical sensing method which only requires mixing of the sensor, base, and carboxylic acid, and can be easily adapted by any laboratory interested in chirality analysis. Interestingly, a similar ICD effect was observed with corresponding nickel complex **5** but not with cobalt analogue **6**.

**Figure 2:**
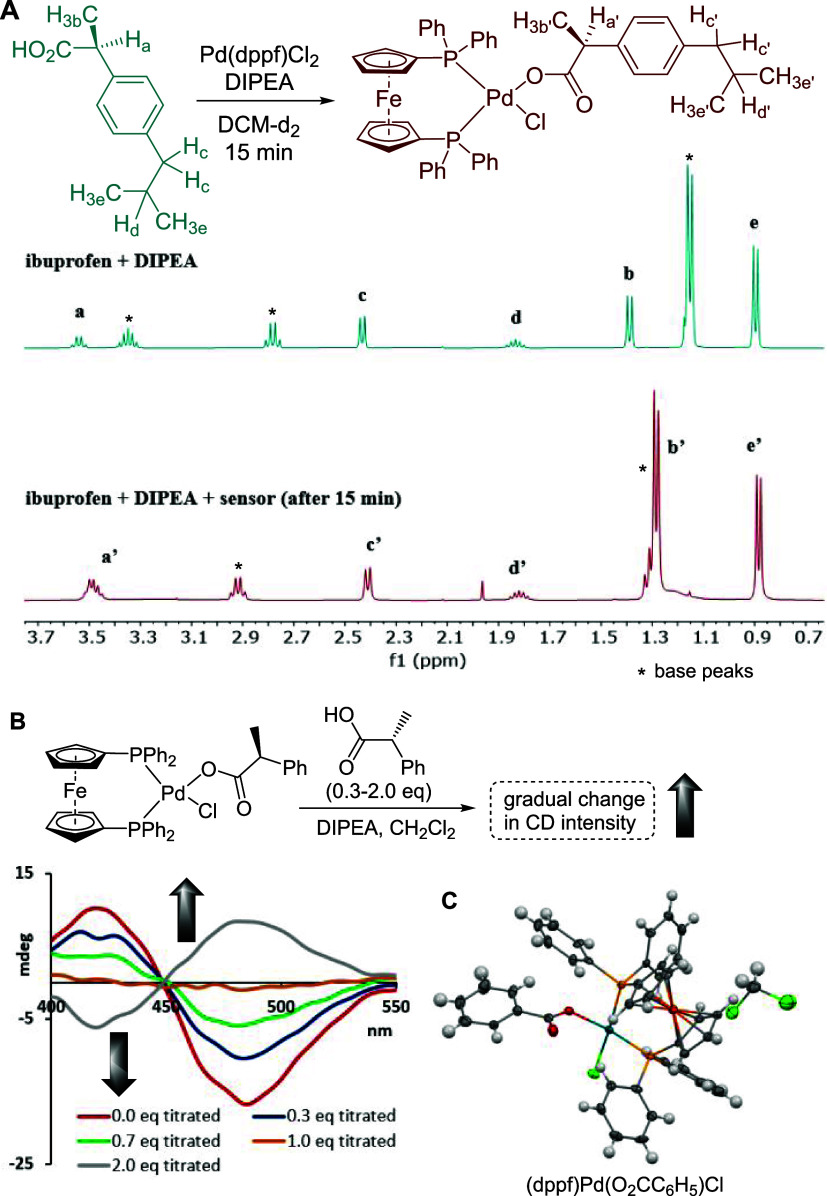
Mechanistic studies. ^1^H NMR reaction monitoring with ibuprofen (A), CD titration experiments with both enantiomers of 2-phenylpropanoic acid (B), and X-ray analysis (ellipsoid contour 50% probability) of a benzoate complex obtained from **4** (C).

We continued investigating the mechanistic underpinnings and experimental parameters of the molecular sensing assay. ^1^H NMR spectroscopic analysis with ibuprofen, **8**, showed that the carboxylate binding is fast and complete within 15 min as indicated by the characteristic upfield shifts of the methyl and methine protons in **8** (Figure 2).

Titration experiments then revealed that excess of the chiral acid does not change the ICD signal intensity, indicating that the chiroptical sensing is a result of stoichiometric analyte coordination, which was further corroborated by ESI-MS analysis (SI). To exclude the possibility of carboxylate- or chloride-bridged dimers or higher supramolecular aggregates that would also consist of equimolar sensor and analyte amounts, we decided to pursue crystallographic analysis. Unfortunately, all attempts to form single crystals with coordination complexes derived from ibuprofen or other analytes and sensor **4** were unsuccessful, presumably because of the high rotational freedom of these acids, which impedes efficient packing into a crystal lattice. We therefore resorted to using silver benzoate to abstract one chloride, which was thus easily removed as AgCl prior to the crystal growth experimentation, and to introduce a rather rigid acid scaffold instead. This approach proved successful, and we were able to grow a single crystal suitable for X-ray crystallography, which is in agreement with our MS analysis and CD titration experiments.^55^ Altogether, these studies suggest that only one chloride is replaced from **4** and that the CD induction originates from the formation of a mononuclear 1:1 coordination complex.

As expected, no CD effect was observed in the absence of base, which is required for carboxylate formation and subsequent metal coordination. Finally, we set out to determine whether the analyte binding to the palladium(II) center is reversible. Titration of increasing amounts of (*R*)-2-phenylpropanoic acid into a solution of the palladium complex originally formed from the (*S*)-enantiomer and **4** showed that the ICD maximum continuously decreases and even reverses when the former is added in excess. Having established that carboxylate coordination is a dynamic process, we considered the possibility of using a chiral Pd complex for NMR spectroscopic enantioselective analysis. However, efforts with ((*R*)-BINAP)PdCl_2_ as a chiral NMR solvating agent under similar conditions did not show any sign of carboxylic acid enantiodifferentiation.

**Figure 3:**
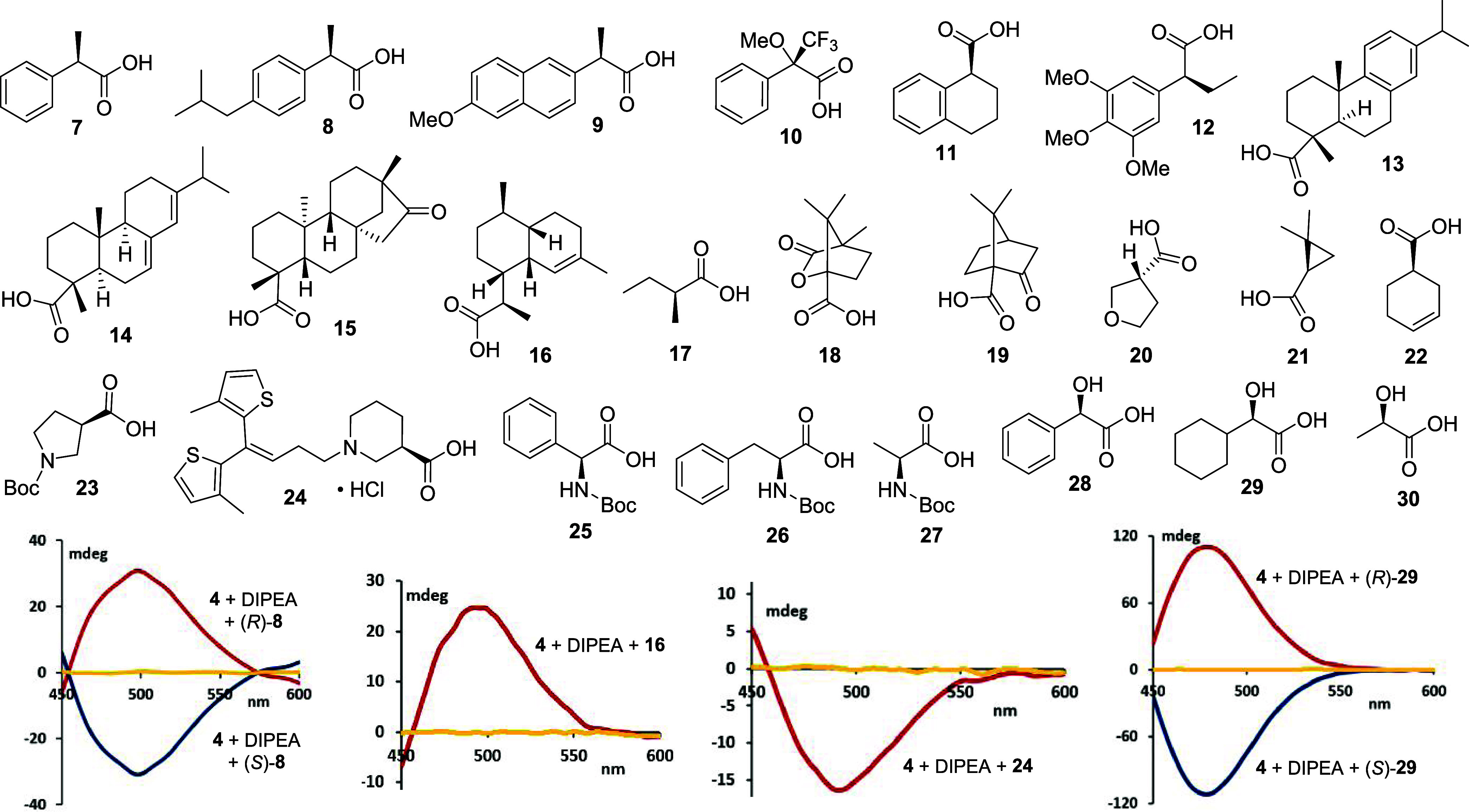
Structures of carboxylic acids tested in this study and selected ICD spectra obtained with **4** (red or blue) or in the absence of **4** (yellow). See the SI for details.

The chiroptical induction obtained with (*S*)-ibuprofen and **4** was then further optimized by screening bases and solvents (SI). We found that Et_3_N, diisopropylethylamine, and sodium *tert*-butoxide give the same ICD signals albeit with varying intensities while K_2_CO_3_, 2,6-lutidine, and pyridine were ineffective. Diisopropylethylamine was chosen for all of the following experiments because it favors strong signal induction under relatively mild conditions. The testing of various combinations of CH_2_Cl_2_, THF, ACN, or MeOH as reactions and diluting solvents showed that the strongest chiroptical responses can be obtained when the assay components are first mixed in CH_2_Cl_2_ and then diluted with MeOH to the desired CD analysis concentration. With this protocol in hand, we were able to evaluate the sensing scope of our assay. For this task, we chose a large selection of chiral carboxylic acids **7**–**30** ranging from the purely aliphatic scaffolds **17** and **21** to analytes carrying aromatic groups, e.g., **10** and **11**, as well as drugs such as naproxen, **9**, and tiagabine, **24**, and natural products like dehydroabietic acid, **13**, abietic acid, **14**, isosteviol, **15**, and dihydroartemisinic acid, **16** (Figure 3). The sensor was also successfully applied to several amino acids **23**–**27**, hydroxy acids **28**–**30**, and other multifunctional compounds such as **12**, **18**, and **19**. Without exception, we observed far red-shifted CD maxima appearing between 450 and 500 nm, while the chiral carboxylic acids remained CD-silent in this area in the absence of the sensor (SI). As is typically the case with small molecule sensors like the palladium complex **4**, the sign and intensity of the CD signal induced upon binding of an analyte is strongly determined by the proximity of its chirality center to the binding site. Enantioselective sensing of compounds exhibiting more distant stereocenters is often not possible or gives significantly decreased CD effects. This trend is apparent by comparison of the decreasing ICD effects obtained by chiroptical sensing of (*R*)-2-phenylbutanoic acid and (*R*)-3-phenylbutanoic acid (see the SI). To this end, the ICDs measured upon binding of **20**–**24** to **4** are quite remarkable and demonstrate the sensitivity of this assay, as these compounds represent some of the most challenging targets by exhibiting remote chirality. However, a comparison of the ICDs obtained with **13**–**15** carrying multiple stereocenters reveals that chiroptical effects originating from the most proximate chirality center dominate those from more distant asymmetric carbon atoms. These three rigid scaffolds share the same absolute configuration at the chirality center adjacent to the acid group, but the stereochemistry at the more remote carbons in compound **15** differs from **13** and **14**. Nevertheless, they all generate a negative CD response upon coordination to **4**, which reveals a somewhat intuitive distance-dependent priority rule, indicating that sensing of diastereomeric scaffolds may be outside the scope of this assay as the more remote stereocenters play significantly diminished roles and can therefore not be effectively differentiated.

To demonstrate the utility of our CD sensing method, we prepared ten samples containing ibuprofen or 1,2,3,4-tetrahydronaphthalene-1-carboxylic acid in various enantiomeric ratios ([*R*]:[*S*]) and overall concentrations ([*R*] + [*S*]). These were then subjected to our chiroptical assay with sensor **4** following a simple protocol that allows determination of both *er* and total acid concentration solely by a series of four CD measurements, i.e., without additional UV or fluorescence sensing and without a calibration curve as previously reported from our laboratory.^38^ This method is only briefly described herein using the first sample containing enantioenriched (*S*)-ibuprofen with an enantiomeric composition of 65 (*S*):35 (*R*) at 35.0 mM as an example. To four 100.0 μL aliquots of this sample were added varying amounts of **4** and an amine base, and the reaction volumes were adjusted to 2.5 mL using CH_2_Cl_2_. These solutions were stirred for 15 min, and CD analysis was performed after diluting 250.0 μL aliquots with 2.0 mL of MeOH. The experimentally obtained CD amplitudes at 490 nm were plotted against the concentrations that **4** would have been at the original sample without the two dilution steps. Linear regression analysis using the CD amplitudes determined in the region of excess analyte over **4** showed a linear increase (blue line). A horizontal line parallel to the *x*-axis representing the range where the CD amplitude is stagnant because the sensor is in excess of the carboxylic acid analyte was also obtained (red line). Importantly, this line shows the maximum ICD signal obtainable with this sample, and the same value would always be measured as long as **4** is present in excess.

The *x*-value at the intersection of these two lines reveals the original concentration of the carboxylic acid in Sample #1 (keeping the sample dilution protocol described above in mind) as 32.9 mM. With the concentration of the analyte in hand, the enantiomeric composition was then calculated by comparing the *y*-axis value (in millideg) with the ICD value that an enantiopure reference would produce at that concentration. This gave an enantiomeric ratio of 65.5:34.5. The absolute configuration of the major ibuprofen enantiomer was determined from the signs of the observed CD signals. We would like to point out that this requires comparison with a reference sample of known absolute configuration when using **4** as the sensor. The determination of the absolute configuration based on induced CD effects is not a trivial task. If a reference sample is not available, it should be conducted either with additional computational analysis or in conjunction with another experimental technique unless the relationship between the sign of the induced Cotton effect and the three-dimensional analyte structure can be generally rationalized based on exciton-coupled CD effects.^56^

This graphical analysis method was applied to all ten samples, and the results are shown in Table 1. To determine the error margins of the concentration and *er* determinations originating from the maximum CD induction deviations observed, the mdeg values were varied by 0.5 mdeg for all four measurements of Sample #1. An averaged (maximum) deviation of 2.08 mM, 6.3% (3.8 mM, 11.6%), was calculated by this analysis. The *er* value on the other hand changed only slightly. The averaged (maximum) *er* deviation calculated was 66.1:33.9, which corresponds to 2.7% (67.0:33.0 or 6.5%). Generally, the comprehensive concentration and *er* sensing analysis occur with good accuracy and error margins that are similar to previously reported methods.^16^

**Table 1:**
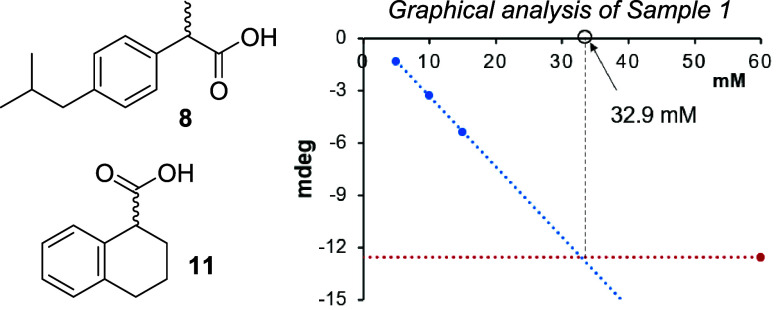
Chirality Sensing of Carboxylic Acid Samples with **4^a^**

	actual composition		CD sensing results	
sample #	conc (mM)	*er* (*S:R*)	conc (mM)	*er* (*S:R*)
1	35.0	65.0:35.0	32.9	65.5:34.5
2	40.0	70.0:30.0	40.3	71.0:29.0
3	45.0	60.0:40.0	43.7	67.0:33.0
4	30.0	95.0:5.0	33.0	90.5:9.5
5	38.0	80.0:20.0	33.0	84.0:16.0
6	35.0	20.0:80.0	36.7	21.0:79.0
7	25.0	65.0:35.0	20.0	72.5:27.5
8	30.0	70.0:30.0	32.0	75.0:25.0
9	28.0	10.0:90.0	26.0	5.5:94.5
10	25.0	25.0:75.0	28.9	22.5:77.5

a Samples 1–5 contained **8**; 6–10 contained **11**. See the SI for details.

## Conclusions

In summary, we have developed a practical chiroptical carboxylic acid sensing method that generates strong CD signals in the visible light region. The molecular recognition is based on the formation of a well-defined equimolar metal complex obtained by the coordination of a carboxylate molecule to 1,1′-bis(diphenylphosphino)ferrocenepalladium dichloride in the presence of a mild base. This ligand substitution process is complete within a few minutes and coincides with the induction of quantifiable CD maxima that occur between 450 and 500 nm. The usefulness of this approach, which is operationally simple and broadly applicable to a variety of acids, including important drugs, natural products, amino acids, and hydroxy acids, was demonstrated with the chiroptical determination of the enantiomeric composition and concentration of ten samples. In addition to the broad application scope and long-wavelength CD inductions, perhaps the most noteworthy advantage over previously reported methods is that this assay allows concentration and *er* analysis solely based on CD measurements. All components needed are commercially available, and this chiroptical sensing method can be readily adapted by any laboratory interested in chirality analysis. The mixing protocol can be executed under air, and it is compatible with generally available high-throughput experimentation equipment and multiwell CD plate readers if parallel analysis of hundreds of samples is desirable.

## Data Availability

The data underlying this study are available in the published article and its Supporting Information.

## Supporting Information Available

Assay development; optimization; mechanistic studies; and analytical details. NMR and CD and MS spectra and crystallographic information (PDF)

## References

[ref1] Piovesana S.; Samperi R.; Laganà A.; Bella M. Determination of Enantioselectivity and Enantiomeric Excess by Mass Spectrometry in the Absence of Chiral Chromatographic Separation: An Overview. Chem. - Eur. J. 2013, 19, 11478–11494. 10.1002/chem.201300233. 23940005

[ref2] Leung D.; Kang S. O.; Anslyn E. V. Rapid Determination of Enantiomeric Excess: A Focus on Optical Approaches. Chem. Soc. Rev. 2012, 41, 448–479. 10.1039/C1CS15135E. 21892514

[ref3] Pu L. Fluorescence of Organic Molecules in Chiral Recognition. Chem. Rev. 2004, 104, 1687–1716. 10.1021/cr030052h. 15008630

[ref4] Shcherbakova E. G.; Minami T.; Brega V.; James T. D.; Anzenbacher P. Jr. Determination of Enantiomeric Excess in Amine Derivatives with Molecular Self-Assemblies. Angew. Chem., Int. Ed. 2015, 54, 7130–7133. 10.1002/anie.201501736. 25925816

[ref5] Akdeniz A.; Minami T.; Watanabe S.; Yokoyama M.; Ema T.; Anzenbacher P. Jr. Determination of enantiomeric excess of carboxylates by fluorescent macrocyclic sensors. Chem. Sci. 2016, 7, 2016–2022. 10.1039/C5SC04235F. 29899926 PMC5968554

[ref6] Sonstrom R. E.; Neill J. L.; Mikhonin A. V.; Doetzer R.; Pate B. H. Chiral Analysis of Pantolactone with Molecular Rotational Resonance Spectroscopy. Chirality 2022, 34, 114–125. 10.1002/chir.23379. 34698412

[ref7] Tielmann P.; Boese M.; Luft M.; Reetz M. T. A Practical High-throughput Screening System for Enantioselectivity by Using FTIR Spectroscopy. Chem. - Eur. J. 2003, 9, 3882–3887. 10.1002/chem.200304885. 12916113

[ref8] Berova N.; Di Bari L.; Pescitelli G. Application of Electronic Circular Dichroism in Configurational and Conformational Analysis of Organic Compounds. Chem. Soc. Rev. 2007, 36, 914–931. 10.1039/b515476f. 17534478

[ref9] Berova N.; Pescitelli G.; Petrovic A. G.; Proni G. Probing Molecular Chirality by CD-sensitive Dimeric Metalloporphyrin Hosts. Chem. Commun. 2009, 5958–5980. 10.1039/b909582a. 19809616

[ref10] Wolf C.; Bentley K. W. Chirality Sensing Using Stereodynamic Probes with Distinct Electronic Circular Dichroism Output. Chem. Soc. Rev. 2013, 42, 5408–5424. 10.1039/c3cs35498a. 23482984

[ref11] Gholami H.; Chakraborty D.; Zhang J.; Borhan B. Absolute Stereochemical Determination of Organic Molecules through Induction of Helicity in Host–Guest Complexes. Acc. Chem. Res. 2021, 54, 654–667. 10.1021/acs.accounts.0c00650. 33428849

[ref12] Prabodh A.; Wang Y.; Sinn S.; Albertini P.; Spies C.; Spuling E.; Yang L.-P.; Jiang W.; Brase S.; Biedermann F. Fluorescence Detected Circular Dichroism (FDCD) for Supramolecular Host–guest Complexes. Chem. Sci. 2021, 12, 9420–9431. 10.1039/D1SC01411K. 34349916 PMC8278969

[ref13] De Dios S. M. R.; Tiwari V. K.; McCune C. D.; Dhokale R. A.; Berkowitz D. B. Biomacromolecule-Assisted Screening for Reaction Discovery and Catalyst Optimization. Chem. Rev. 2022, 122, 13800–13880. 10.1021/acs.chemrev.2c00213. 35904776 PMC12088691

[ref14] Herrera B. T.; Pilicer S. L.; Anslyn E. V.; Joyce L. A.; Wolf C. Optical Analysis of Reaction Yield and Enantiomeric Excess. A New Paradigm Ready for Prime Time. J. Am. Chem. Soc. 2018, 140, 10385–10401. 10.1021/jacs.8b06607. 30059621

[ref15] Hassan D. S.; Kariapper F. S.; Lynch C. C.; Wolf C. Accelerated Asymmetric Reaction Screening with Optical Assays. Synthesis 2022, 54, 2527–2538. 10.1055/a-1754-2271.

[ref16] Formen J. S. S. K.; Howard J. R.; Anslyn E. V.; Wolf C. Circular Dichroism Sensing: Strategies and Applications. Angew. Chem., Int. Ed. 2024, 63, e202400767 10.1002/anie.202400767. 38421186

[ref17] Wezenberg S. J.; Salassa G.; Escudero-Adan E. C.; Benet-Buchholz J.; Kleij A. W. Effective Chirogenesis in a Bis(metallosalphen) Complex Through Host–Guest Binding with Carboxylic Acids. Angew. Chem., Int. Ed. 2011, 50, 713–716. 10.1002/anie.201004957. 20967813

[ref18] Joyce L. A.; Maynor M. S.; Dragna J. M.; da Cruz G. M.; Lynch V. M.; Canary J. W.; Anslyn E. V. A Simple Method for the Determination of Enantiomeric Excess and Identity of Chiral Carboxylic Acids. J. Am. Chem. Soc. 2011, 133, 13746–13752. 10.1021/ja205775g. 21780788 PMC3179184

[ref19] De los Santos Z. A.; Yusin G.; Wolf C. Enantioselective Sensing of Carboxylic Acids with a Bis(urea)oligo(phenylene)ethynylene Foldamer. Tetrahedron 2019, 75, 1504–1509. 10.1016/j.tet.2019.01.070.

[ref20] Osawa K.; Tagaya H.; Kondo S. I. Induced Circular Dichroism of Achiral Cyclic Bisurea via Hydrogen Bonds with Chiral Carboxylates. J. Org. Chem. 2019, 84, 6623–6630. 10.1021/acs.joc.9b00073. 30913877

[ref21] Bentley K. W.; Proano D.; Wolf C. Chirality Imprinting and Direct Asymmetric Reaction Screening Using a Stereodynamic Brønsted/Lewis Acid Receptor. Nat. Commun. 2016, 7, 12539 10.1038/ncomms12539. 27549926 PMC4996974

[ref22] Huang X.; Wang X.; Quan M.; Yao H.; Ke H.; Jiang W. Biomimetic Recognition and Optical Sensing of Carboxylic Acids in Water by Using a Buried Salt Bridge and the Hydrophobic Effect. Angew. Chem., Int. Ed. 2021, 60, 1929–1935. 10.1002/anie.202012467. 33089632

[ref23] Huang X.; Rickman B. H.; Borhan B.; Berova N.; Nakanishi K. Zinc Porphyrin Tweezer in Host-guest Complexation: Determination of Absolute Configurations of Diamines, Amino Acids, and Amino Alcohols by Circular Dichroism. J. Am. Chem. Soc. 1998, 120, 6185–6186. 10.1021/ja973539e. 11931101

[ref24] Barcena H. S.; Holmes A. E.; Zahn S.; Canary J. W. Redox Inversion of Helicity in Propeller-Shaped Molecules Derived from S-Methyl Cysteine and Methioninol. Org. Lett. 2003, 5, 709–711. 10.1021/ol0275217. 12605496

[ref25] Kim H.; So S. M.; Yen C. P.; Vinhato E.; Lough A. J.; Hong J. I.; Kim H. J.; Chin J. Highly Stereospecific Generation of Helical Chirality by Imprinting with Amino Acids: A Universal Sensor for Amino Acid Enantiopurity. Angew. Chem., Int. Ed. 2008, 47, 8657–8660. 10.1002/anie.200803116. 18846521

[ref26] Joyce L. A.; Canary J. W.; Anslyn E. V. Enantio- and Chemoselective Differentiation of Protected α-Amino Acids and β-Homoamino Acids with a Single Copper(II) Host. Chem. - Eur. J. 2012, 18, 8064–8069. 10.1002/chem.201103592. 22592912 PMC3416025

[ref27] Scaramuzzo F. A.; Licini G.; Zonta C. Determination of Amino Acid Enantiopurity and Absolute Configuration: Synergism between Configurationally Labile Metal-Based Receptors and Dynamic Covalent Interactions. Chem. - Eur. J. 2013, 19, 16809–16813. 10.1002/chem.201302721. 24173828

[ref28] Bentley K. W.; Nam Y. G.; Murphy J. M.; Wolf C. Chirality Sensing of Amines, Diamines, Amino Acids, Amino Alcohols, and α-Hydroxy Acids with a Single Probe. J. Am. Chem. Soc. 2013, 135, 18052–18055. 10.1021/ja410428b. 24261969

[ref29] Biedermann F.; Nau W. M. Noncovalent Chirality Sensing Ensembles for the Detection and Reaction Monitoring of Amino Acids, Peptides, Proteins, and Aromatic Drugs. Angew. Chem., Int. Ed. 2014, 53, 5694–5699. 10.1002/anie.201400718. 24719396

[ref30] Badetti E.; Wurst K.; Licini G.; Zonta C. Multimetallic Architectures from the Self-assembly of Amino Acids and Tris(2-pyridylmethyl)amine Zinc(II) Complexes: Circular Dichroism Enhancement by Chromophores Organization. Chem. - Eur. J. 2016, 22, 6515–6518. 10.1002/chem.201600480. 26888188

[ref31] De los Santos Z. A.; Wolf C. Chiroptical Asymmetric Reaction Screening via Multicomponent Self-Assembly. J. Am. Chem. Soc. 2016, 138, 13517–13520. 10.1021/jacs.6b08892. 27696842

[ref32] Thanzeel F. Y.; Wolf C. Substrate-Specific Amino Acid Sensing Using a Molecular D/L-Cysteine Probe for Comprehensive Stereochemical Analysis in Aqueous Solution. Angew. Chem., Int. Ed. 2017, 56, 7276–7281. 10.1002/anie.201701188. 28508583

[ref33] Thanzeel F. Y.; Sripada A.; Wolf C. Quantitative Chiroptical Sensing of Free Amino acids, Biothiols, Amines and Amino Alcohols with an Aryl Fluoride Probe. J. Am. Chem. Soc. 2019, 141, 16382–16387. 10.1021/jacs.9b07588. 31564090

[ref34] Nelson E.; Formen J. S. S. K.; Wolf C. Rapid Organocatalytic Chirality Analysis of Amines, Amino Acids, Alcohols, Amino Alcohols and Diols with Achiral Iso(thio)cyanate Probes. Chem. Sci. 2021, 12, 8784–8790. 10.1039/D1SC02061G. 34257878 PMC8246279

[ref35] Li B.; Zhang J.; Li L.; Chen G. A Rapid and Sensitive Method for Chiroptical Sensing of α-Amino Acids via Click-like Labeling with o-Phthalaldehyde and p-Toluenethiol. Chem. Sci. 2021, 12, 2504–2508. 10.1039/D0SC05749E. PMC8179345 34164017

[ref36] Hassan D. S.; De los Santos Z. A.; Brady K. G.; Murkli S.; Isaacs L.; Wolf C. Chiroptical Sensing of Amino Acids, Amines, Amino Alcohols, Alcohols and Terpenes with π-Extended Acyclic Cucurbiturils. Org. Biomol. Chem. 2021, 19, 4248–4253. 10.1039/D1OB00345C. 33885685

[ref37] Formen J. S. S. K.; Wolf C. Chiroptical Switching and Quantitative Chirality Sensing with (Pseudo)halogenated Quinones. Angew. Chem., Int. Ed. 2021, 60, 27031–27038. 10.1002/anie.202111542. 34679202

[ref38] Sripada A.; Thanzeel F. Y.; Wolf C. Unified Sensing of the Concentration and Enantiomeric Composition of Chiral Compounds with an Achiral Probe. Chem 2022, 8, 1734–1749. 10.1016/j.chempr.2022.03.008.

[ref39] Wu X.; Chen X.-X.; Song B.-N.; Huang Y.-J.; Li Z.; Chen Z.; James T. D.; Jiang Y.-B. Induced Helical Chirality of Perylenebisimide Aggregates Allows for Enantiopurity Determination and Differentiation of α-Hydroxy Carboxylates by Using Circular Dichroism. Chem. - Eur. J. 2014, 20, 11793–11799. 10.1002/chem.201402627. 25078854

[ref40] Peng R.; Lin L.; Cao W.; Guo J.; Liu X.; Feng X. A Racemic N,N ′-Dioxide-iron(III) Complex Chemosensor for Determination of Enantiomeric Excess, Concentration and Identity of Hydroxy Carboxylic Acids with Circular Dichroism and Fluorescence Responses. Tetrahedron Lett. 2015, 56, 3882–3885. 10.1016/j.tetlet.2015.04.101.

[ref41] Bentley K. W.; Zhang P.; Wolf C. Miniature High-Throughput Chemosensing of Yield, Ee and Absolute Configuration From Crude Reaction Mixtures. Sci. Adv. 2016, 2, e1501162 10.1126/sciadv.1501162. 26933684 PMC4758738

[ref42] Puri M.; Ferry V. E. Circular Dichroism of CdSe Nanocrystals Bound by Chiral Carboxylic Acids. ACS Nano 2017, 11, 12240–12246. 10.1021/acsnano.7b05690. 29164858

[ref43] De los Santos Z. A.; Lynch C. C.; Wolf C. Optical Chirality Sensing with an Auxiliary-Free Earth-Abundant Cobalt Probe. Angew. Chem., Int. Ed. 2019, 58, 1198–1202. 10.1002/anie.201811761. 30500091

[ref44] De los Santos Z. A.; Joyce L. A.; Sherer E. C.; Welch C. J.; Wolf C. Optical Chirality Sensing with a Stereodynamic Aluminum Biphenolate Probe. J. Org. Chem. 2019, 84, 4639–4645. 10.1021/acs.joc.8b01301. 30019902

[ref45] Lynch C. C.; De los Santos Z. A.; Wolf C. Chiroptical Sensing of Unprotected Amino Acids, Hydroxy acids, Amino Alcohols, Amines and Carboxylic Acids with Metal Salts. Chem. Commun. 2019, 55, 6297–6300. 10.1039/C9CC02525A. 31089587

[ref46] Thanzeel F. Y.; Balaraman K.; Wolf C. Quantitative Chirality and Concentration Sensing of Alcohols, Diols, Hydroxy Acids, Amines and Amino Alcohols using Chlorophosphite Sensors in a Relay Assay. Angew. Chem., Int. Ed. 2020, 59, 21382–21386. 10.1002/anie.202005324. 32762103

[ref47] Begato F.; Penasa R.; Licini G.; Zonta C. Chiroptical Enhancement of Chiral Dicarboxylic Acids from Confinement in a Stereodynamic Supramolecular Cage. ACS Sens. 2022, 7, 1390–1394. 10.1021/acssensors.2c00038. 35472260 PMC9150167

[ref48] Moor S. R.; Howard J. R.; Herrera B. T.; McVeigh M. S.; Marini F.; Keating-Clay A. T.; Anslyn E. V. Determination of Enantiomeric Excess and Diastereomeric Excess via Optical Methods. Application to α-Methyl-α-hydroxy-carboxylic Acids. Org. Chem. Front. 2023, 10, 1386–1392. 10.1039/D2QO01444K. 37636898 PMC10456989

[ref49] Yang Q.; Olmsted C.; Borhan B. Absolute Stereochemical Determination of Chiral Carboxylic Acids. Org. Lett. 2002, 4, 3423–3426. 10.1021/ol026527t. 12323034

[ref50] Tanasova M.; Anyika M.; Borhan B. Sensing Remote Chirality: Stereochemical Determination of β-, γ-, and δ-Chiral Carboxylic Acids. Angew. Chem., Int. Ed. 2015, 54, 4274–4278. 10.1002/anie.201410371. 25684753

[ref51] Akdeniz A.; Mosca L.; Minamia T.; Anzenbacher P. Jr. Sensing of Enantiomeric Excess in Chiral Carboxylic Acids. Chem. Commun. 2015, 51, 5770–5773. 10.1039/C5CC00376H. 25720499

[ref52] Proni G.; Pescitelli G.; Huang X.; Quaraishi N. Q.; Nakanishi K.; Berova N. Configurational Assignment of α-Chiral Carboxylic Acids by Complexation to Dimeric Zn-Porphyrin: Host-Guest Structure, Chiral Recognition and Circular Dichroism. Chem. Commun. 2002, 1590–1591. 10.1039/b204554k 12170796

[ref53] Huang L.; Hu C.; Wang Y. Chirality Sensing of Chiral Carboxylic Acids by a Ureido-Linked Zinc Bisporphyrinate. Chem. - Asian J. 2024, 19, e202400359 10.1002/asia.202400359. 38744672

[ref54] Pilicer S. L.; Dragna J. M.; Garland A.; Welch C. J.; Anslyn E. V.; Wolf C. High Throughput Determination of Enantiopurity by Microplate Circular Dichroism. J. Org. Chem. 2020, 85, 10858–10864. 10.1021/acs.joc.0c01395. 32705865

[ref55] Neo Y. C.; Yeo J. S. L.; Low P. M. N.; Chien S. W.; Mak T. C. W.; Vittal J. J.; Hor T. S. A. Isolation and structural characterization of some stable Pd(II) carboxylate complexes supported by 1,1′-bis(diphenylphosphino) ferrocene (dppf). J. Organomet. Chem. 2002, 658, 159–168. 10.1016/S0022-328X(02)01645-5.

[ref56] Polavarapu P. L. Why is it Important to Simultaneously Use More Than One Chiroptical Spectroscopic Method for Determining the Structures of Chiral Molecules?. Chirality 2008, 20, 664–672. 10.1002/chir.20475. 17924421

