# Supplementary material for: Molecular Sensing of Chiral Carboxylic Acid Enantiomers Using CD Inductions in the Visible Light Region
Source: J Org Chem. 2025 Jan 2;90(2):994–1000. doi: 10.1021/acs.joc.4c02055 (PMC11744787; doi:10.1021/acs.joc.4c02055)

Supporting Information

Molecular Sensing of Chiral Carboxylic Acid Enantiomers Using CD Inductions in the Visible Light Region

Jeffrey S. S. K. Formen, Eryn Nelson and Christian Wolf*

Department of Chemistry, Georgetown University, 37th and O St., Washington DC 20057, USA.

Email: cw27@georgetown.edu

**Contents**	**Page**
1. General information	S2
2. Probe development and mechanistic studies	S2
2.1. Sensor screening	S2
2.2. NMR reaction analysis	S6
2.3. Investigation of ^{1}H NMR enantiodifferentiation with ((*R*)-BINAP)$PdCl_2$	S8
2.4. Binding motif analysis	S9
2.5. Dynamic equilibrium titration study	S11
3. Optimization of sensing conditions	S12
3.1. Reaction stoichiometry	S12
3.2. Solvent and base screening	S14
4. Carboxylic acid substrate scope	S15
5. Quantitative carboxylic acid sensing: enantiomeric ratio and total concentration	S33
5.1. Simultaneous concentration and *er* analysis of ibuprofen samples	S33
5.2. Simultaneous concentration and *er* analysis of 1,2,3,4-tetrahydronaphthalene-1-carboxylic acid samples	S38

1. General information

All commercially available chemicals and solvents were used without further purification. NMR spectra were obtained on a Varian at 400 MHz (^{1}H NMR) at room temperature using CD_2Cl_2 or $CDCl_3$ as solvent. Chemical shifts are reported in ppm relative to the solvent peak. CD spectra were collected with a standard sensitivity of 100 mdeg, a data pitch of 1.0 nm, and a bandwidth of 1.0 nm in a continuous scanning mode with a scanning speed of 500 nm/min and a response of 1.0 s (1 cm path length). The data were baseline corrected and smoothed using a binomial equation. Single crystal analysis was performed at 100K using a Siemens platform diffractometer with a graphite monochromated Mo-Kα radiation (λ = 0.71073 Å). Data were integrated and corrected using the APEX 3 program. The structures were solved by direct methods and refined with full-matrix least-square analysis using SHELXL-2019/1 software. Non-hydrogen atoms were refined with anisotropic displacement parameter. Mass spectrometry was performed on a single quadrupole LC/MSD iQ. For reactions that require heating an oil bath was used as the heat source.

2. Probe development and mechanistic studies

2.1. Sensor screening

To a solution of (*R*)-2-phenylpropanoic acid (12.5 mg, 0.08 mmol) and DIPEA (10.3 mg, 0.08 mmol) in 1.0 mL of CH_2Cl_2 was added sensor **1-6** (0.08 mmol in 1.0 mL of CH_2Cl_2). The mixture was allowed to stir for 1 hour, diluted to 0.66-2.65 mM with THF (concentration is indicated under each figure) and subjected to CD analysis. Sensor **4** gave the strongest and most red-shifted CD signal with a maximum of 40 mdeg at 475 nm.

Scheme S1. Chiroptical sensing of (*R*)-2-phenylpropanoic acid with sensors **1-6**.

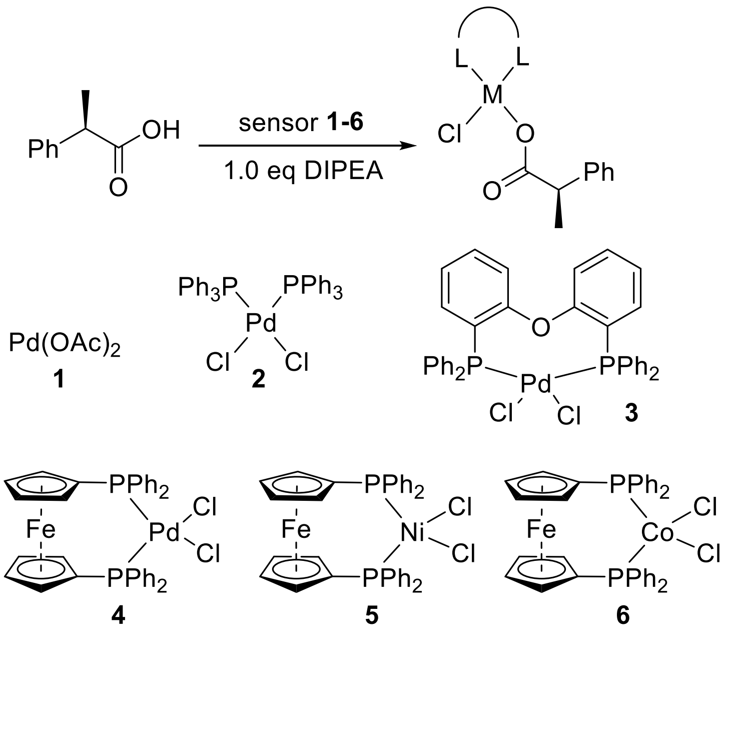

Figure S1. Chiroptical sensing of (*R*)-2-phenylpropanoic acid with sensor **1**.

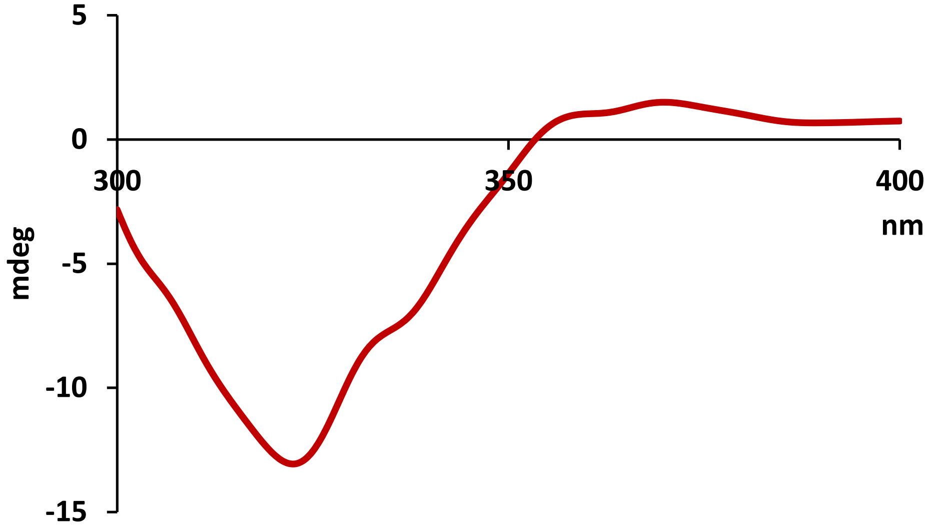

CD measurements were taken at 1.32 mM in THF.

Figure S2. Chiroptical sensing of (*R*)-2-phenylpropanoic acid with sensor **2**.

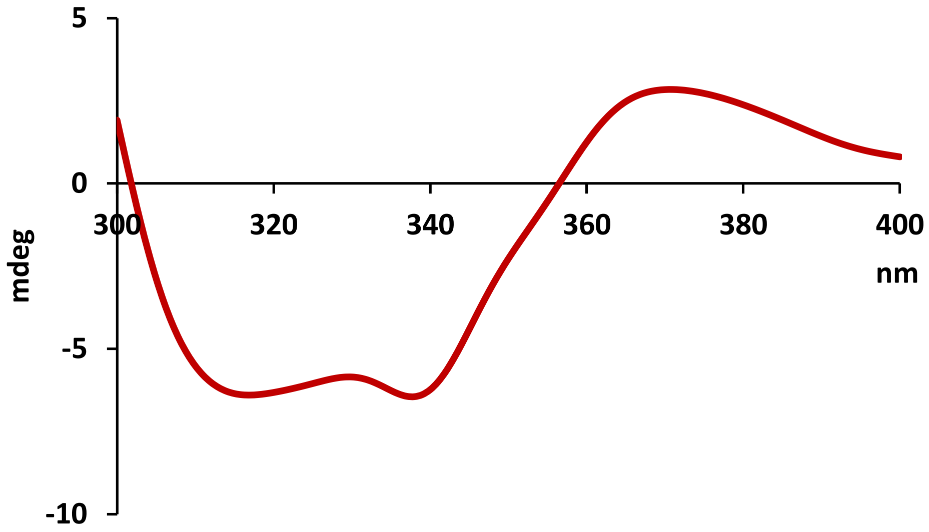

CD measurements were taken at 0.66 mM in THF.

Figure S3. Chiroptical sensing of (*R*)-2-phenylpropanoic acid with sensor **3**.

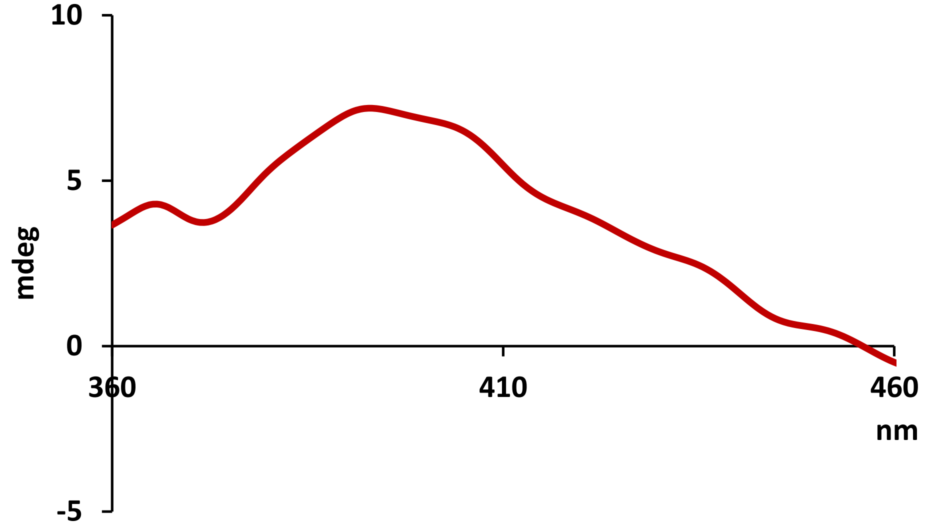

CD measurements were taken at 1.32 mM in THF.

Figure S4. Chiroptical sensing of (*R*)-2-phenylpropanoic acid with sensor **4**.

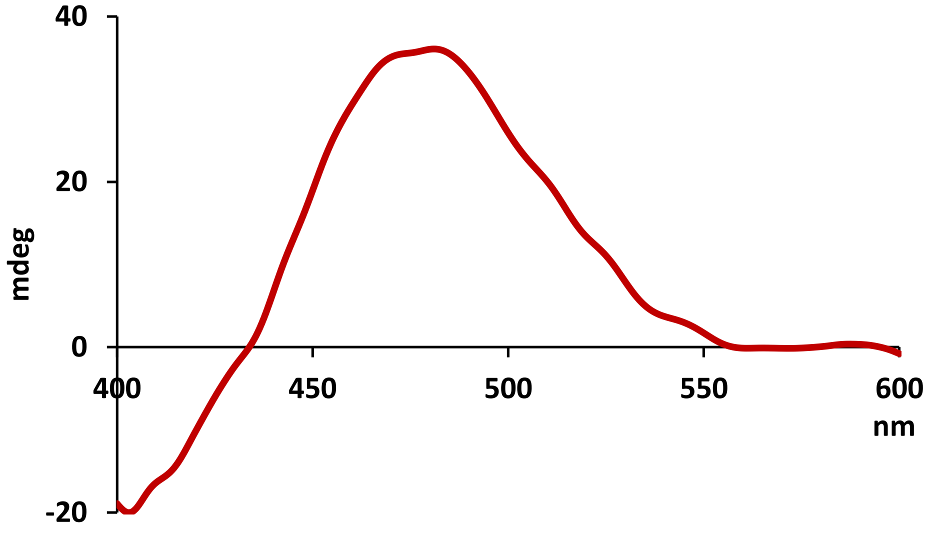

CD measurements were taken at 2.65 mM in THF.

Figure S5. Chiroptical sensing of (*R*)-2-phenylpropanoic acid with sensor **5**.

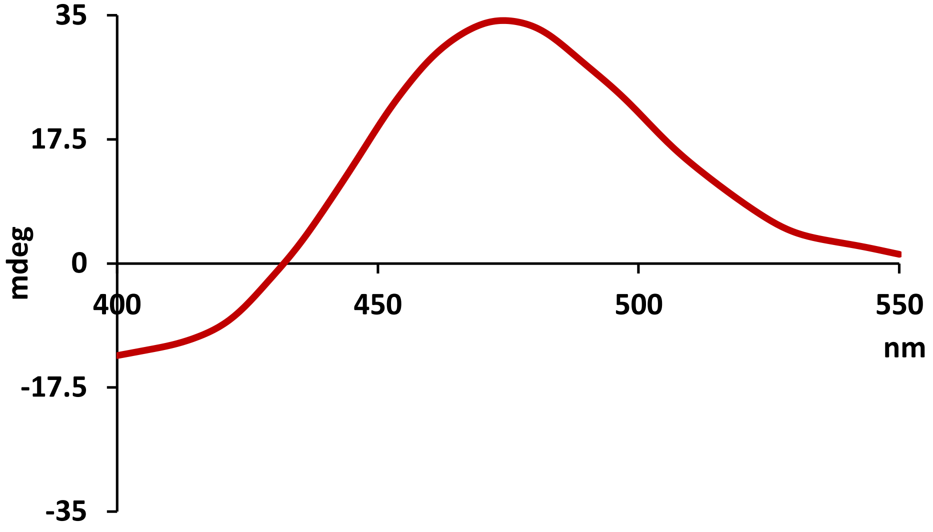

CD measurements were taken at 2.65 mM in THF.

Figure S6. Chiroptical sensing of (*R*)-2-phenylpropanoic acid with sensor **6**.

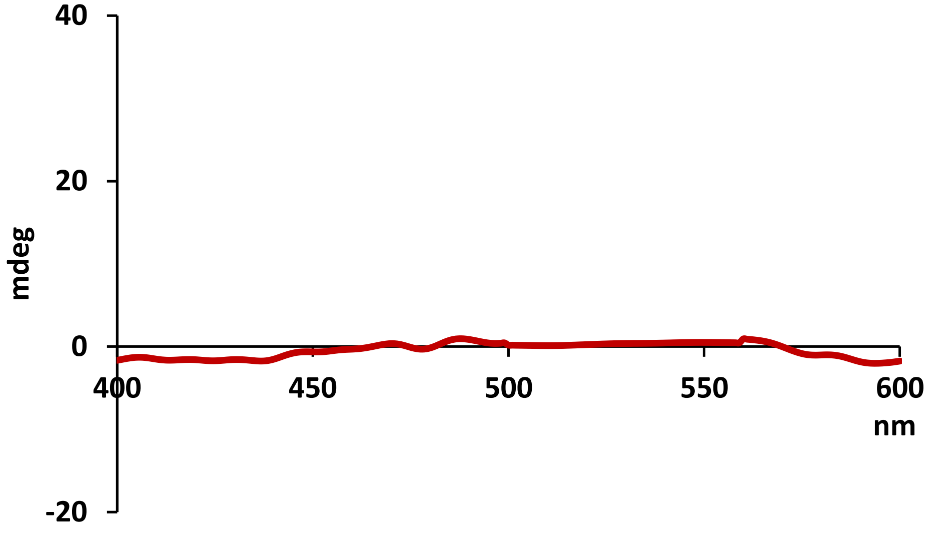

CD measurements were taken at 2.65 mM in THF.

2.2. NMR reaction analysis

(*S*)-Ibuprofen (22.7 mM), DIPEA (22.7 mM), and sensor **4** (22.7 mM) were mixed in 2.2 mL DCM-d$_2$ and the reaction was monitored by ^{1}H NMR. The reaction was complete within 15 minutes.

Scheme S2. NMR analysis of the reaction between (*S*)-ibuprofen and **4**.

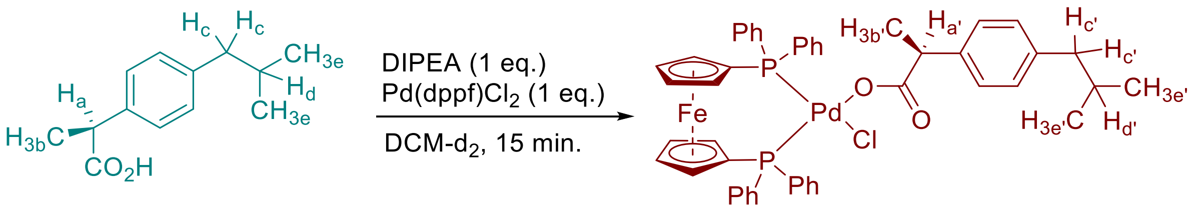

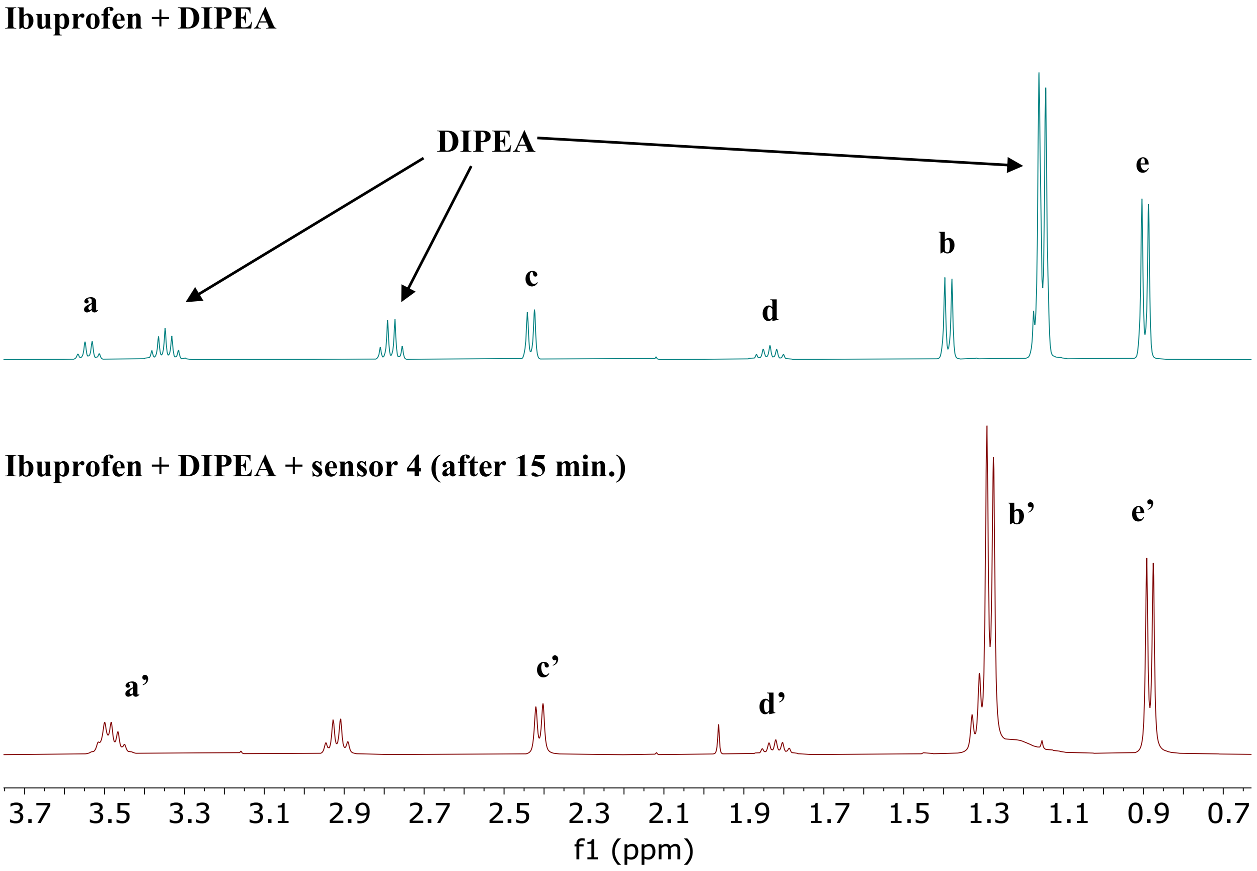

Scheme S3. NMR analysis of the reaction between (*S*)-ibuprofen and **4** (full spectra).

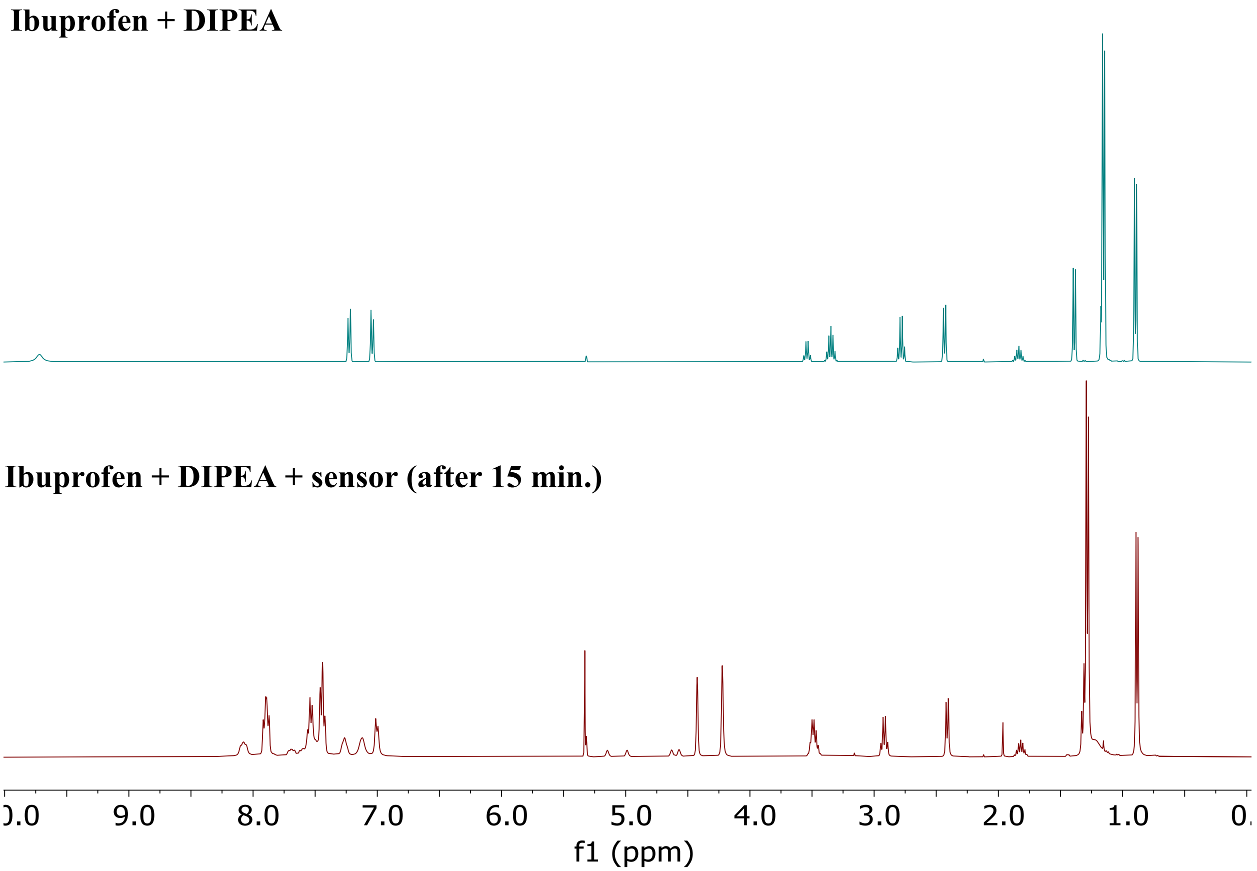

2.3. Investigation of ^{1}H NMR enantiodifferentiation with ((*R*)-BINAP)$PdCl_2$

Racemic 2-phenylpropionic acid (20.0 mg, 0.13 mmol), DIPEA (17.2 mg, 0.13 mmol), and ((*R*)-BINAP)$PdCl_2$ (106.5 mg, 0.13 mmol) were combined in 1.5 mL $CDCl_3$. The mixture was allowed to stir for 1 hour and then subjected to ^{1}H NMR analysis. No sign of resolution of the diastereomeric benzylic protons was observed.

Scheme S4. NMR analysis of the reaction between racemic 2-phenylpropionic acid and ((*R*)-BINAP)$PdCl_2$.

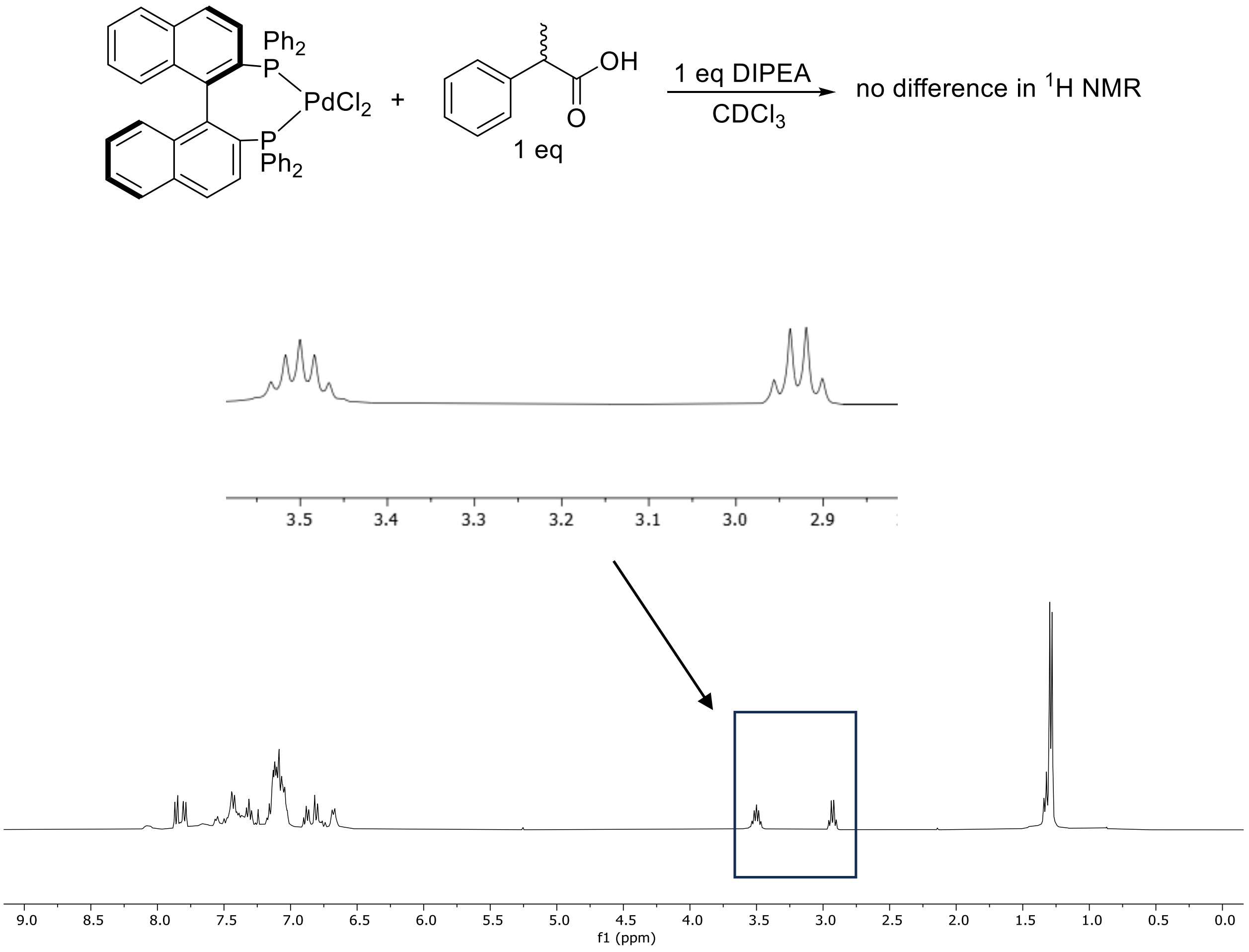

2.4. Binding motif analysis

X-ray crystallography

Attempts to obtain single crystals from chiral acids with sensor **4** were unsuccessful. Due to the highly crystalline nature of **4** and reversibility of the reaction, we only obtained single crystals of **4**. To drive the equilibrium towards product formation, we employed silver benzoate to precipitate and remove AgCl. To a solution of sensor **4** (200.0 mg, 0.24 mmol) in 10.0 mL of CH_2Cl_2 was added silver benzoate (56.1 mg, 0.24 mmol) under nitrogen. After 18 hours under reflux, the mixture was filtered and pentane was carefully layered onto the solution (pentane: CH_2Cl_2, 1:1, v/v) to grow single crystals. X-ray analysis was performed at 100 K using a Siemens platform diffractometer with a graphite monochromated Mo-Kα radiation (λ = 0.71073 Å). Data were integrated and corrected using the APEX 3 program. The structures were solved by direct methods and refined with full-matrix least-square analysis using SHELXL-2019/1 software. Non-hydrogen atoms were refined with anisotropic displacement parameter. Crystal data: $C_{42}H_{35}Cl_3FeO_2P_2Pd$, *M*= 902.24, 0.238 x 0.084 x 0.078 mm^3, triclinic, space group P-1, a= 9.264(3), b= 13.747 (3), c= 15.892(8) Å, Z = 2.

Scheme S5. X-ray crystallographic analysis (ellipsoid contour 50% probability) of the reaction product of **4** and silver benzoate.

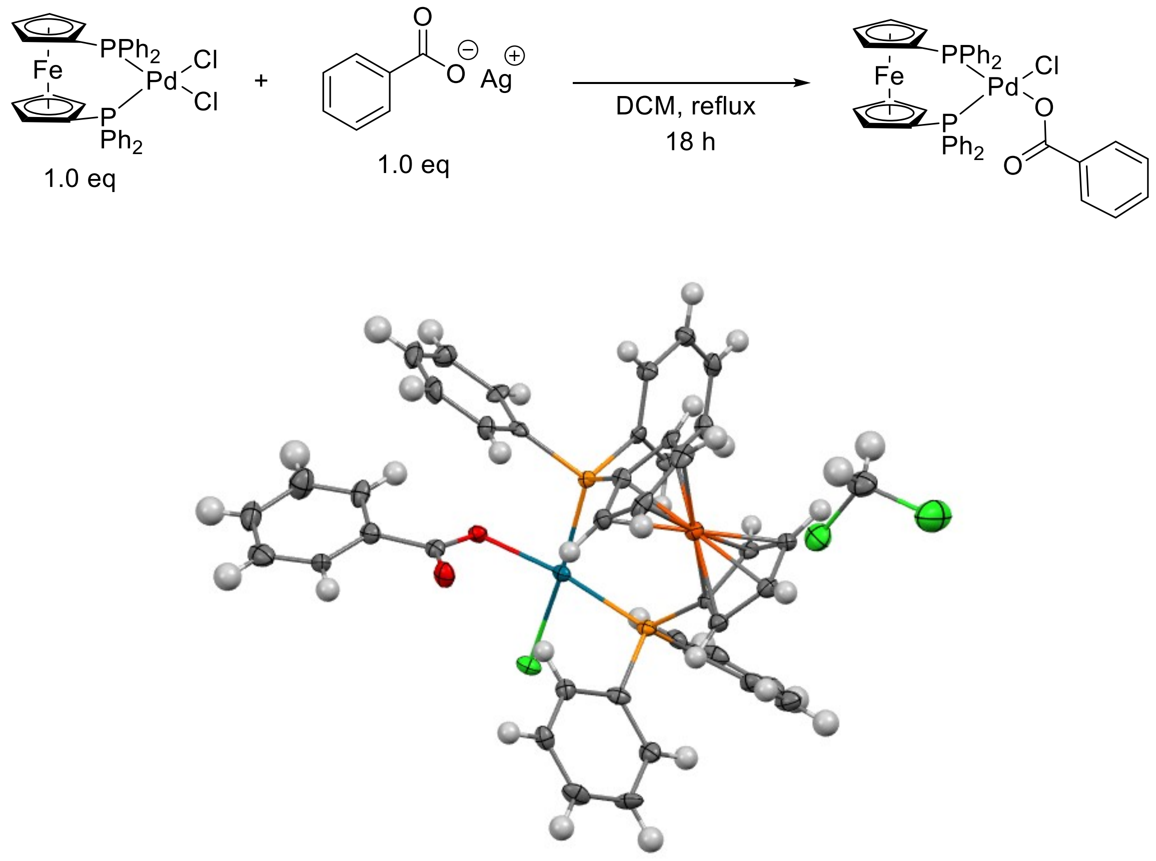

The CCDC number for the crystal reported in this study is 2362916.

Mass spectrometry

To solution of sensor **4** (20.4 mg, 0.025 mmol) in 2.0 mL CH_2Cl_2 were added (*S*)-ibuprofen (5.2 mg, 0.025 mmol) and DIPEA (0.025 mmol). The mixture was allowed to stir for 1 hour. Direct injection into a single quadrupole LC/MSD iQ showed binding of the analyte to sensor **4** in a 1:1 ratio.

Figure S7. ESI-MS analysis of reaction product of **4** and ibuprofen in presence of DIPEA.

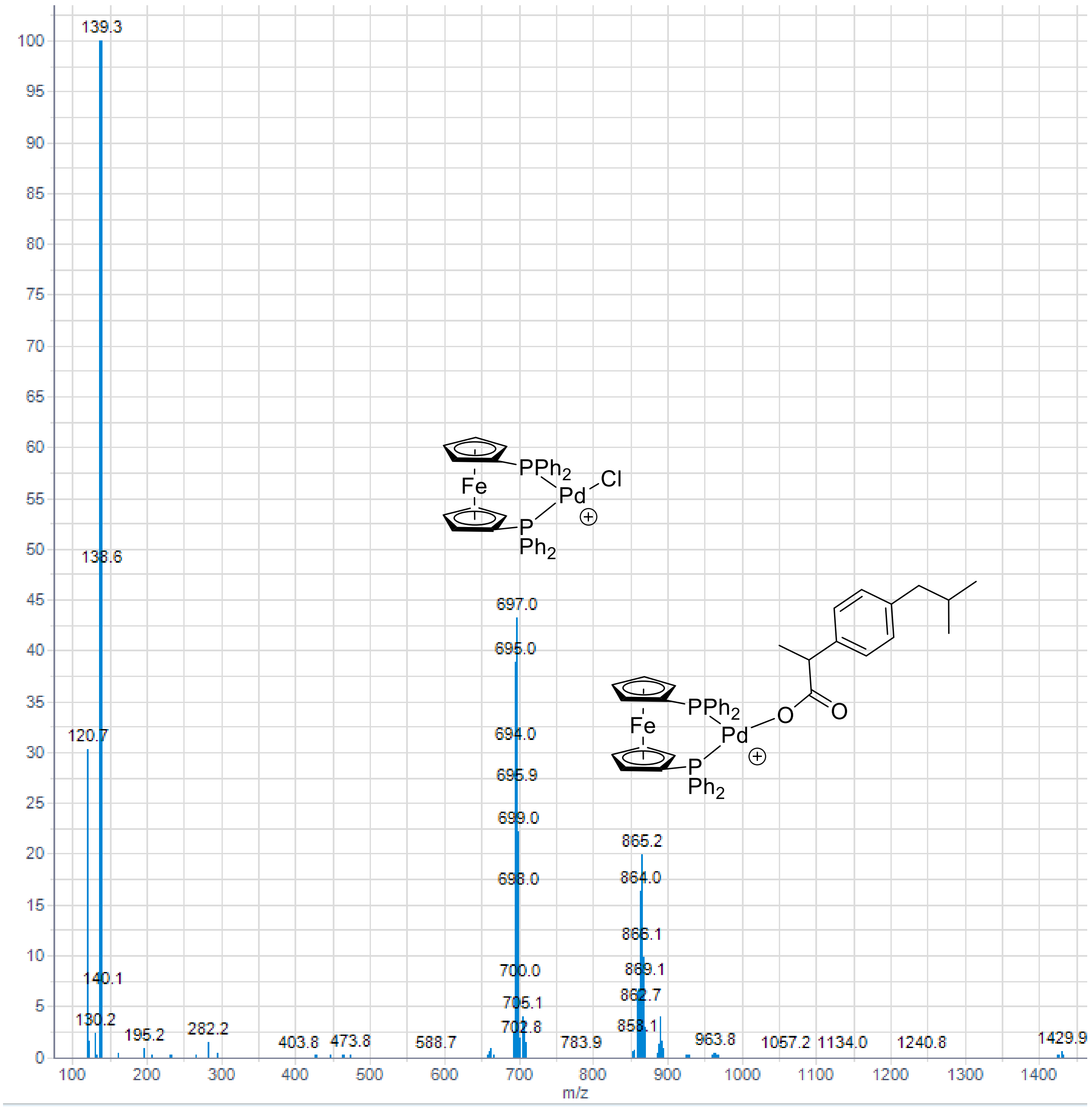

2.5. Dynamic equilibrium titration study

(*S*)-2-Phenylpropionic acid (5.2mg, 0.025 mmol), DIPEA (3.2 mg, 0.025 mmol), and sensor **4** (20.4 mg, 0.025 mmol) were combined in 2.2 mL of CH_2Cl_2. The mixture was allowed to stir for 1 hour after which (*R*)-2-phenylpropionic acid was gradually added in (0.3 eq, 0.7 eq, 1.0 eq, 2.0 eq). After 1 hour the mixtures were subjected to CD analysis. Titration of (*R*)-2-phenylpropionic acid showed a decrease and even reversal of the CD amplitude, demonstrating the dynamic equilibrium of this system.

Scheme S6. Titration of (*R*)-2-phenylpropionic acid portions to (dppf)Pd((*S*)-**7**)Cl

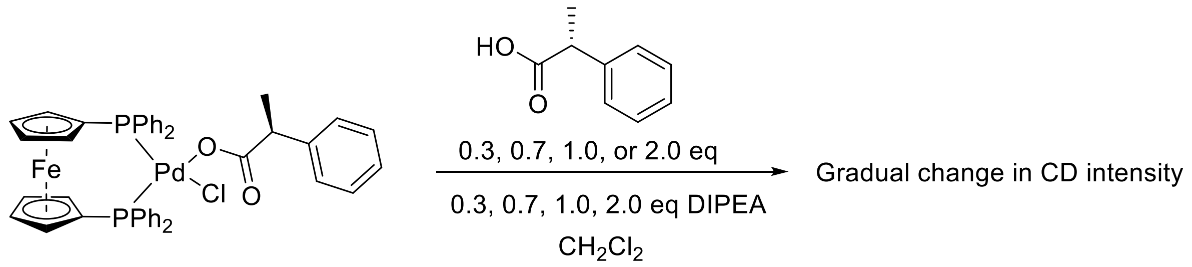

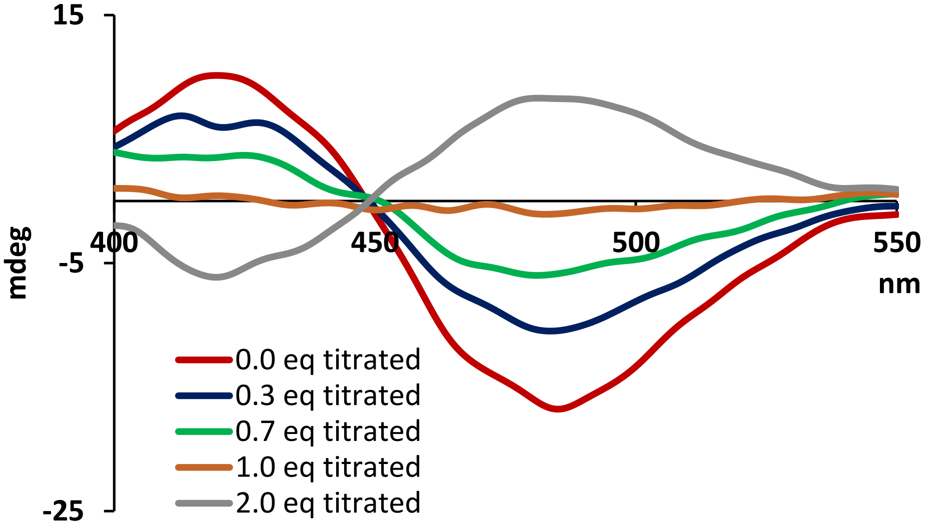

3. Optimization of sensing conditions

3.1. Reaction stoichiometry

To a solution of sensor **4** (20.4 mg, 0.025 mmol) in 2.0 mL CH_2Cl_2 was added either 1.0 or 2.0 eq of (*S*)-ibuprofen and DIPEA. The mixture was allowed to stir for 1 hour, diluted to 2.65 mM in CH_2Cl_2 and subjected to CD analysis. No significant difference in the CD spectra was observed.

Scheme S7. Effect of analyte and base equivalence on CD sensing with sensor **4**.

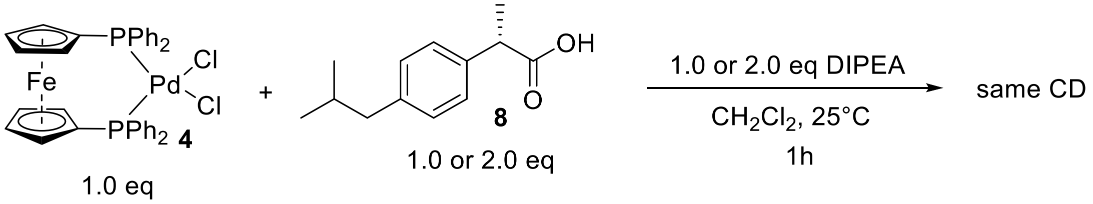

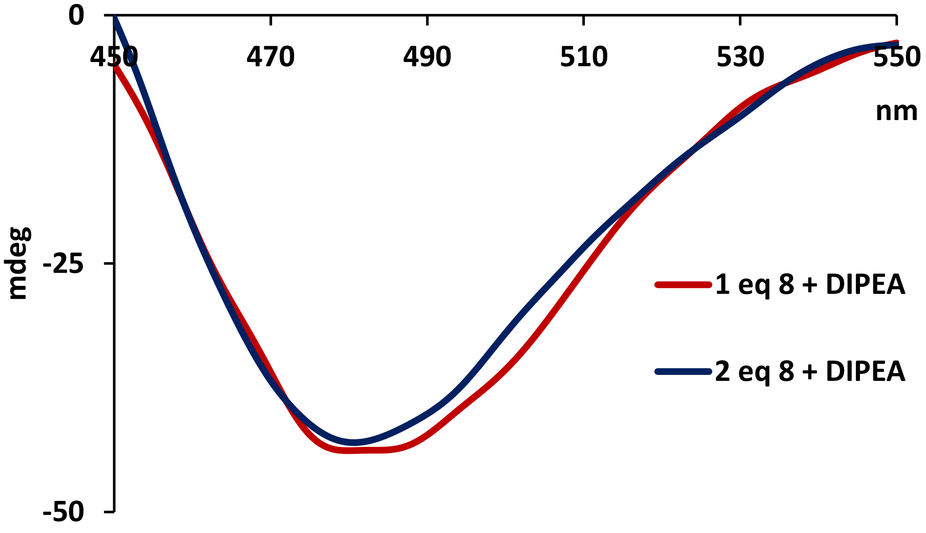

CD measurements were taken at 2.65 mM in CH_2Cl_2.

To a solution of (*S*)-ibuprofen (5.2 mg, 0.025 mmol) and DIPEA (3.2 mg, 0.025 mmol) in 2.0 mL CH_2Cl_2 was added either 1.0 eq or 2.0 eq of sensor **4**. The mixture was allowed to stir for 1 hour, diluted to 2.65 mM in CH_2Cl_2 and subjected to CD analysis. No significant difference in the CD spectra was observed.

Scheme S8. Effect of sensor equivalence on CD induction.

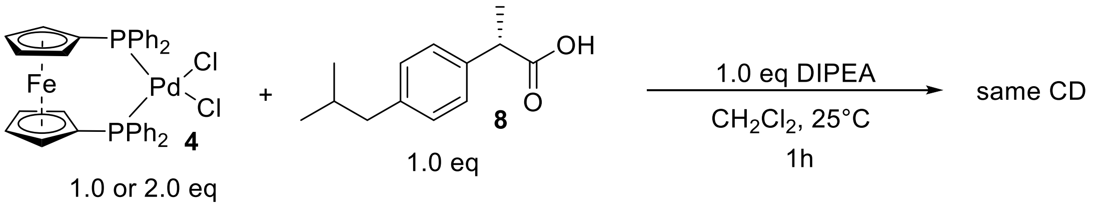

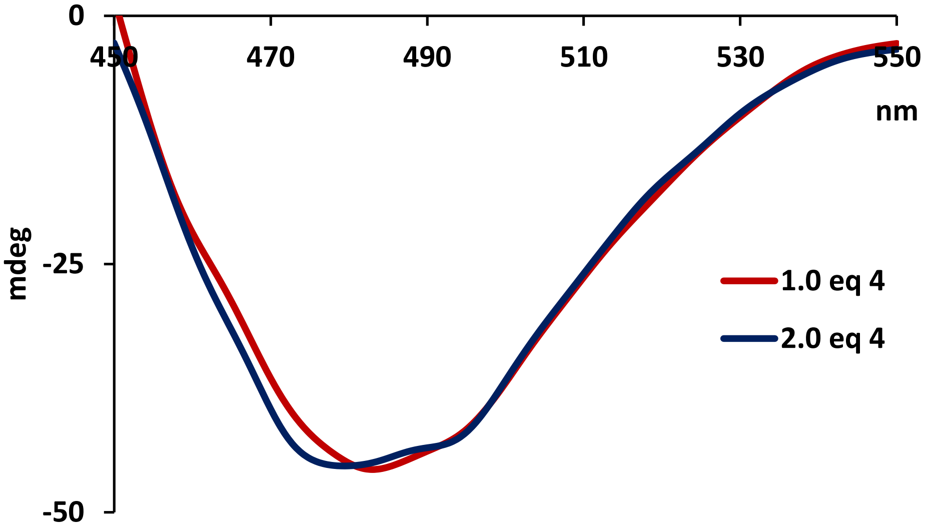

CD measurements were taken at 2.65 mM in CH_2Cl_2.

3.2. Solvent and base screening

To solution of sensor **4** (20.4 mg, 0.025 mmol) in 2.0 mL CH_2Cl_2 was added (*S*)-ibuprofen (5.2 mg, 0.025 mmol) and either pyridine, 2,6-lutidine, K_2CO_3, Et_3N, DIPEA, NaO*t*-Bu (0.025 mmol) or no base. The mixture was allowed to stir for 1 hour and was diluted to 2.65 mM in CH_2Cl_2 and subjected to CD analysis. Induction of CD signals was observed when DIPEA, Et_3N, or NaO*t*-Bu were used. No CD signal induction was observed with pyridine, 2,6-lutidine and K_2CO_3 or in the absence of base.

Scheme S9. Effect of base on the ICD effect with sensor **4**.

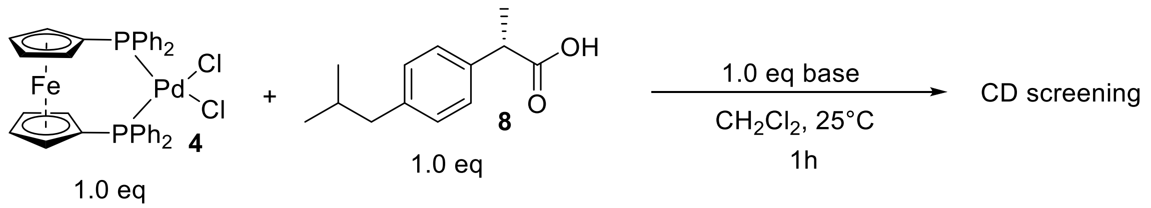

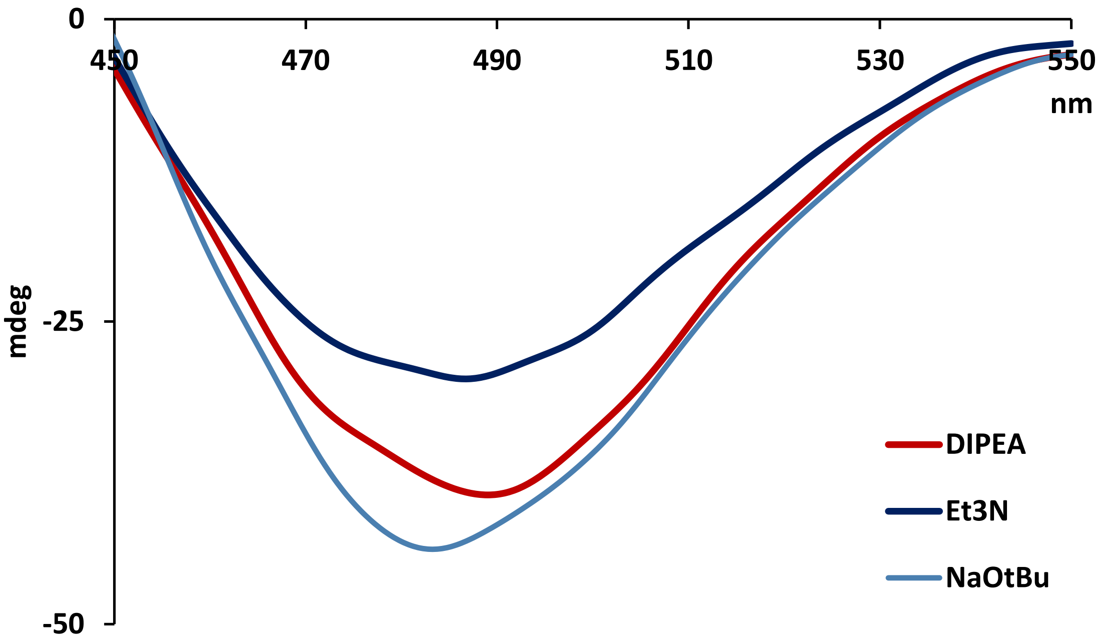

CD measurements were taken at 2.65 mM in CH_2Cl_2.

(*S*)-Ibuprofen (5.2mg, 0.025 mmol), DIPEA (3.2 mg, 0.025 mmol) and sensor **4** (20.4 mg, 0.025 mmol) were dissolved in either 2.5 mL of CH_2Cl_2 or MeOH. The mixtures were allowed to stir for 1 hour and were diluted to 2.65 mM with CH_2Cl_2, THF, ACN, or MeOH for CD analysis. The strongest CD induction was obtained when CH_2Cl_2 was used as the reaction solvent and MeOH as the dilution solvent. All subsequent experiments were thus conducted using CH_2Cl_2 as solvent and MeOH as dilution solvent.

Figure S9. Solvent effect on the CD response of **4** to acid **8**.

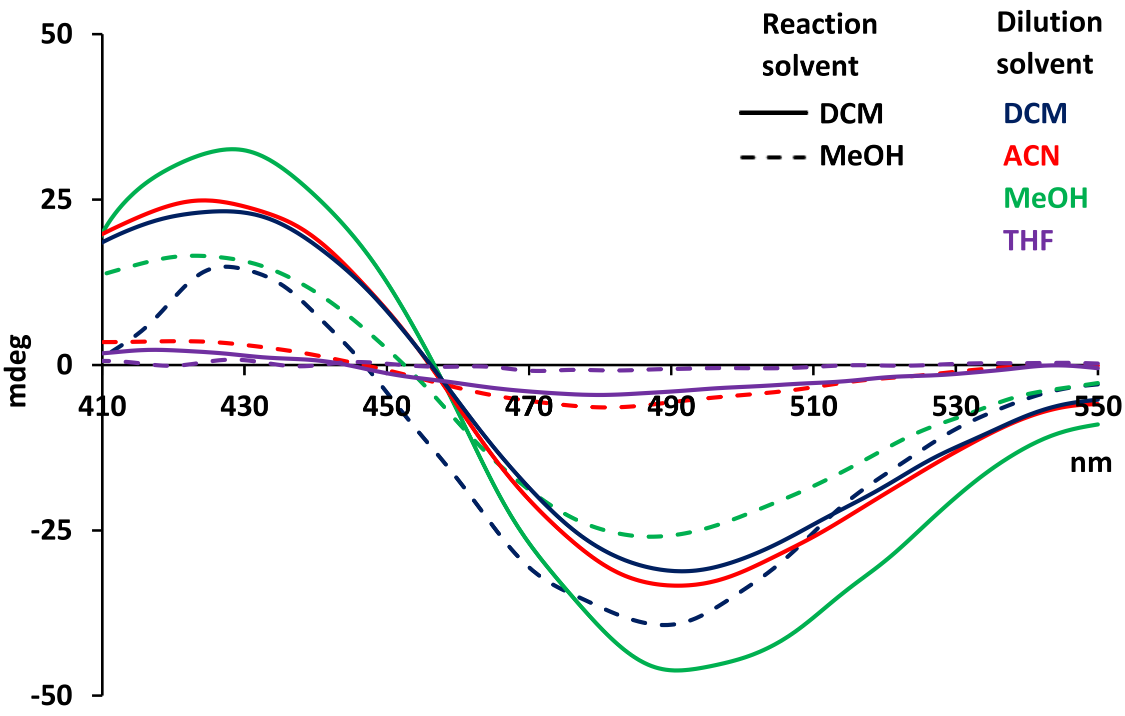

4. Carboxylic acid substrate scope

A solution of a chiral carboxylic acid (**7-30**) (22.7 mM), DIPEA (22.7 mM), and sensor **4** (22.7 mM) in 2.2 mL dichloromethane was stirred for 15 minutes. For acids **24-30**, 2 equivalents of DIPEA (45.4 mM) were used. CD analysis was performed after dilution with methanol to the final concentration indicated under each Figure (150–350 µL of the reaction mixture added to 2.0 mL MeOH). The CD spectra were collected with a standard sensitivity of 100 mdeg, a data pitch of 0.5 nm, and a bandwidth of 1 nm in a continuous scanning mode with a scanning speed of 500 nm/min and a response of 1 s (1 cm path length). The data were baseline corrected and smoothed using a binomial equation.

Scheme S10. Carboxylic acid, amino acid and hydroxy acid scope

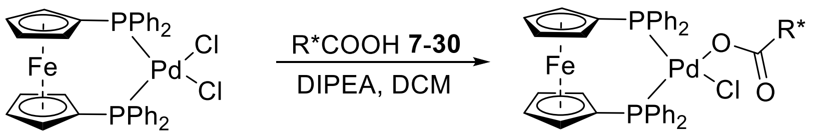

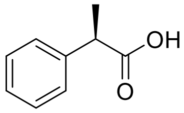
7

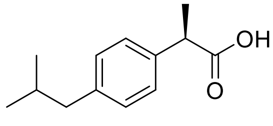
8

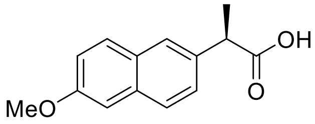
9

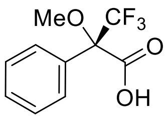
10

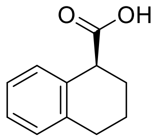
11

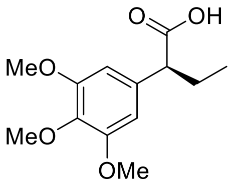
12

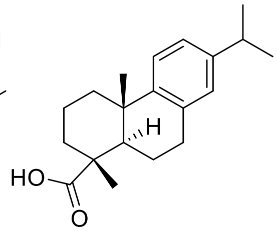
13

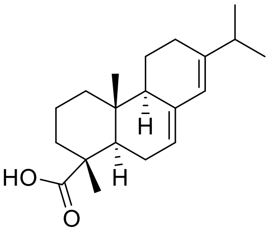
14

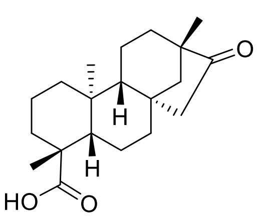
15

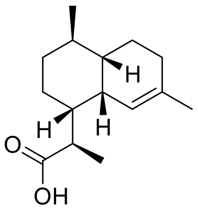
16

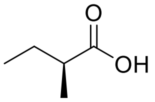
17

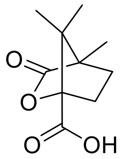
18

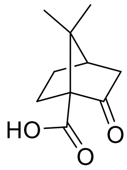
19

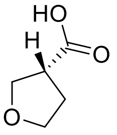
20

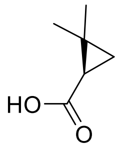
21

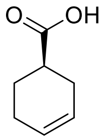
22

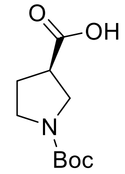
23

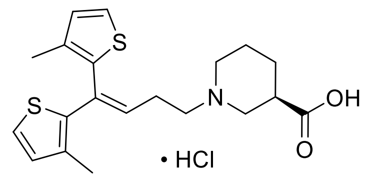
24

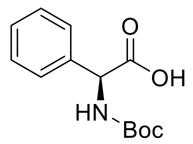
25

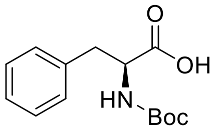
26

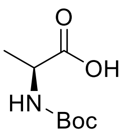
27

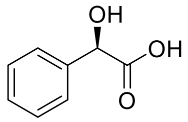
28

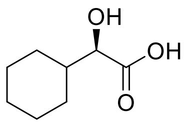
29

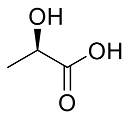
30

Only one enantiomer is shown.

Figure S9. CD spectra obtained by applying sensor **4** to (*R*)-**7** (red) and (*S*)-**7** (blue).

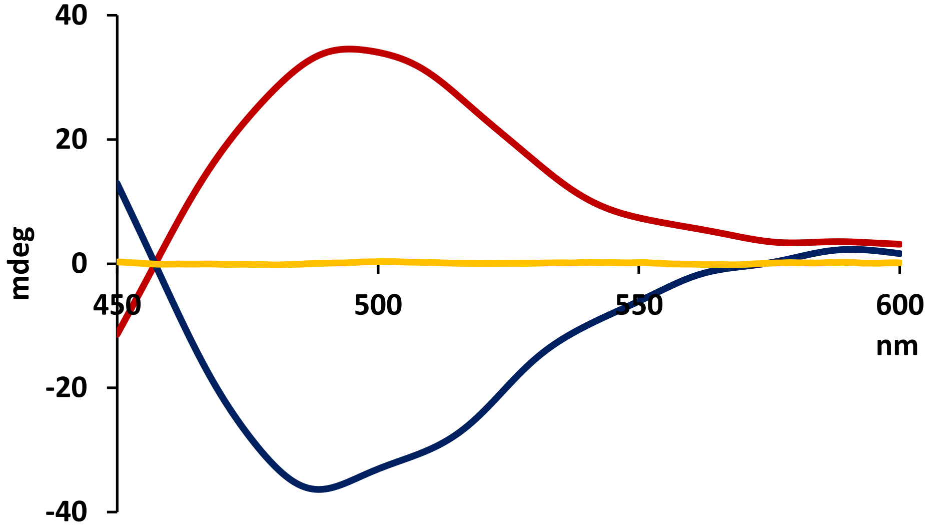

CD measurements were taken at 2.5 mM in MeOH.

Figure S10. CD spectra obtained by applying sensor **4** to (*R*)-**8** (red) and (*S*)-**8** (blue) vs. (*S*)-**8** only (yellow).

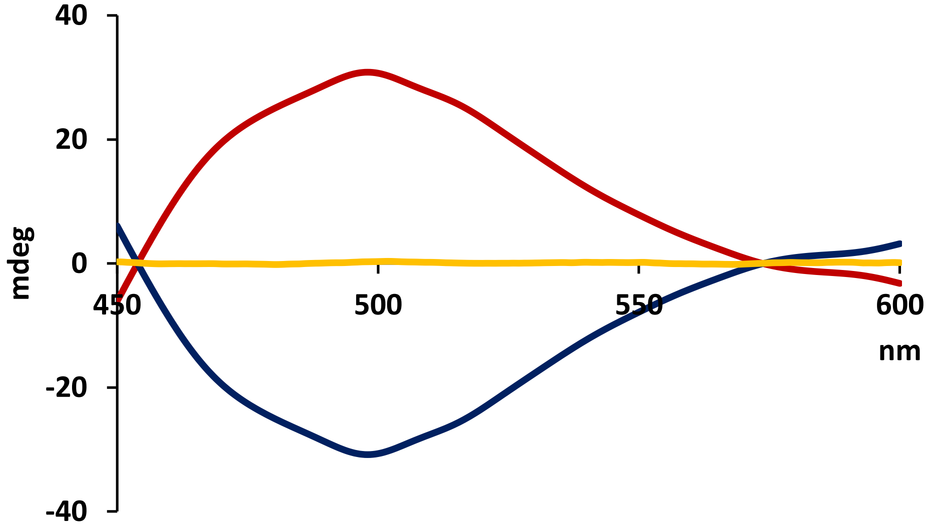

CD measurements were taken at 2.5 mM in MeOH.

Figure S11. CD spectra obtained by applying sensor **4** to (*R*)-**9** (red) and (*S*)-**9** (blue) vs. (*S*)-**9** only (yellow).

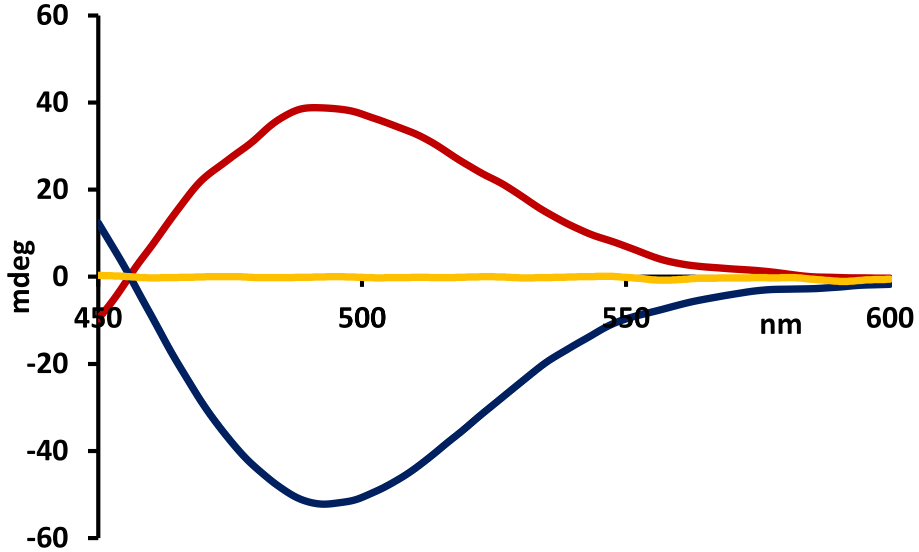

CD measurements were taken at 2.5 mM in MeOH. The enantiopurity of (*R*)-**9** was low (>80% *ee*)

Figure S12. CD spectra obtained by applying sensor **4** to (*R*)-**10** (red) vs. (*R*)-**10** only (yellow).

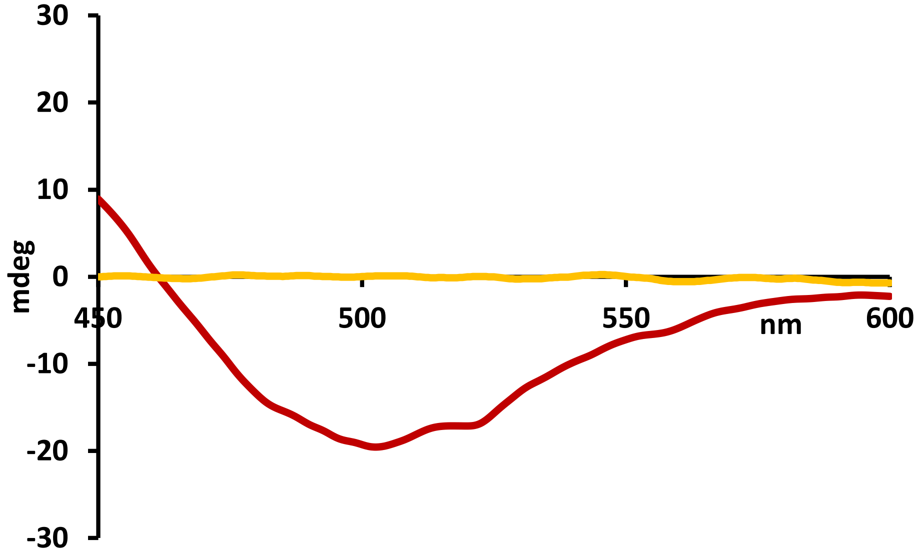

CD measurements were taken at 2.5 mM in MeOH.

Figure S13. CD spectra obtained by applying sensor **4** to (*R*)-**11** (red) and (*S*)-**11** (blue) vs. (*R*)-**11** only (yellow).

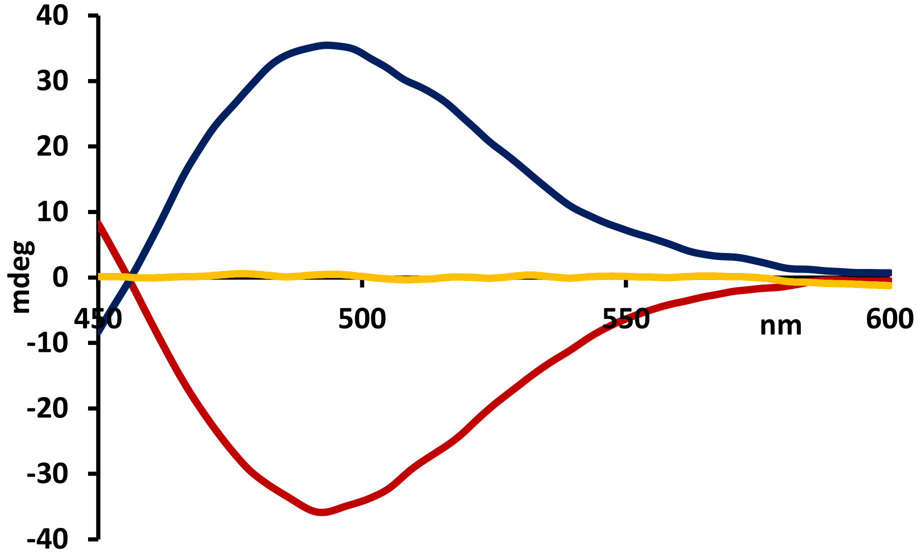

CD measurements were taken at 2.5 mM in MeOH.

Figure S14. CD spectra obtained by applying sensor **4** to (*S*)-**12** (red) vs. (*S*)-**12** only (yellow).

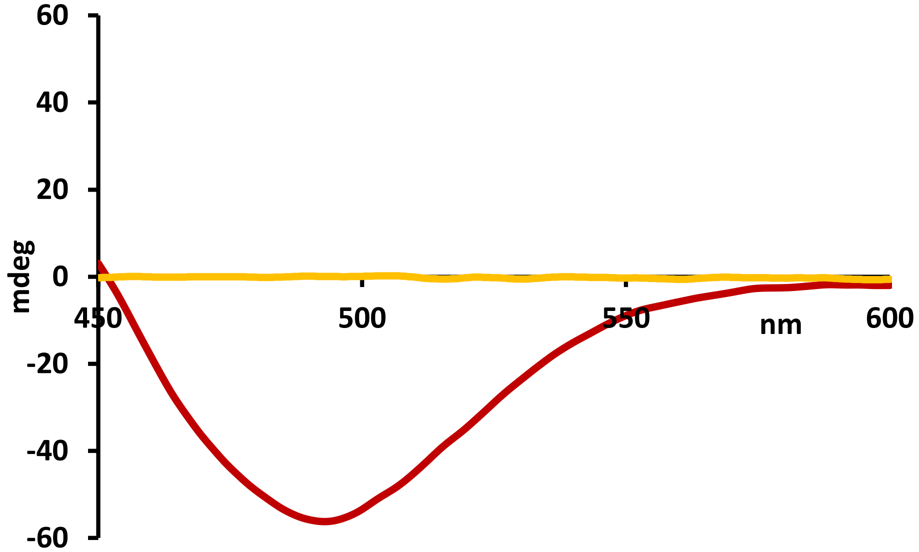

CD measurements were taken at 2.5 mM in MeOH.

Figure S15. CD spectra obtained by applying sensor **4** to dehydroabietic acid (**13**, red) vs. **13** only (yellow).

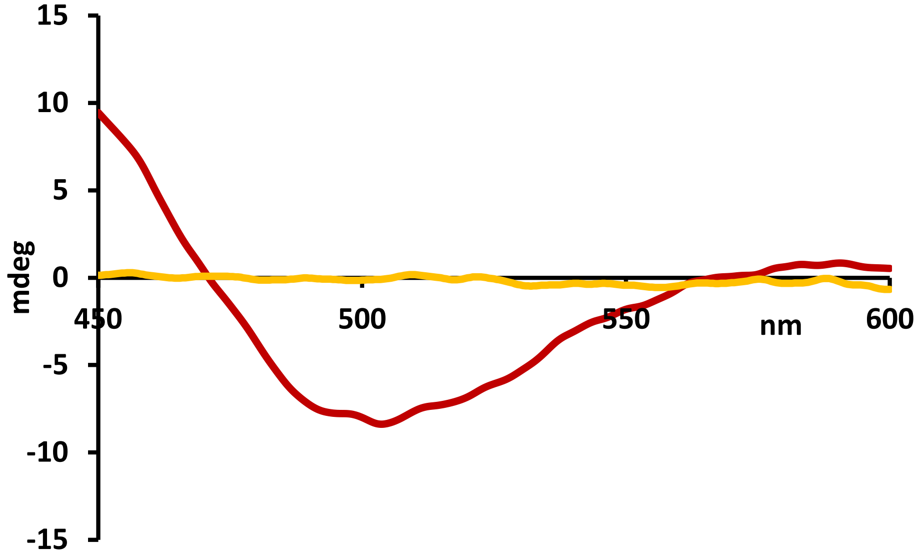

CD measurements were taken at 2.5 mM in MeOH.

Figure S16. CD spectra obtained by applying sensor **4** to abietic acid (**14**, red) vs. **14** only (yellow).

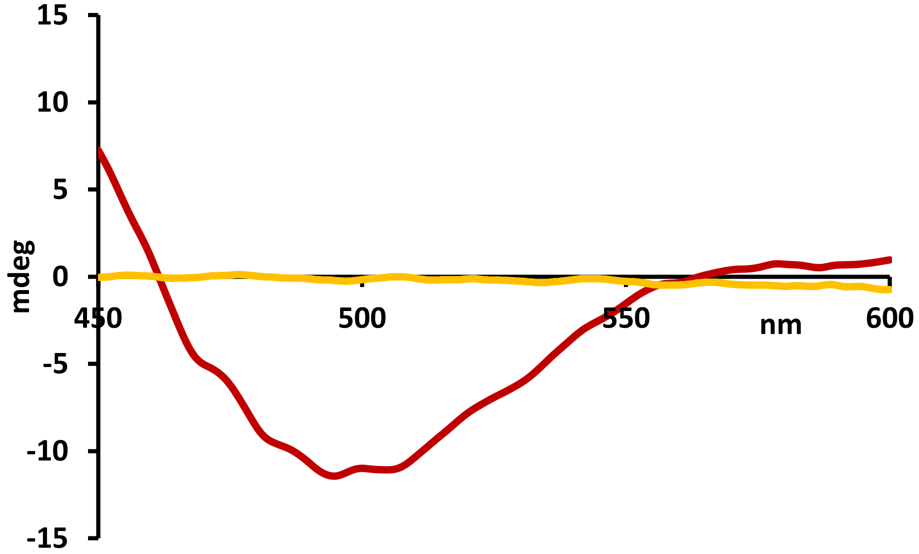

CD measurements were taken at 2.5 mM in MeOH.

Figure S17. CD spectra obtained by applying sensor **4** to isosteviol (**15**, red) vs. **15** only (yellow).

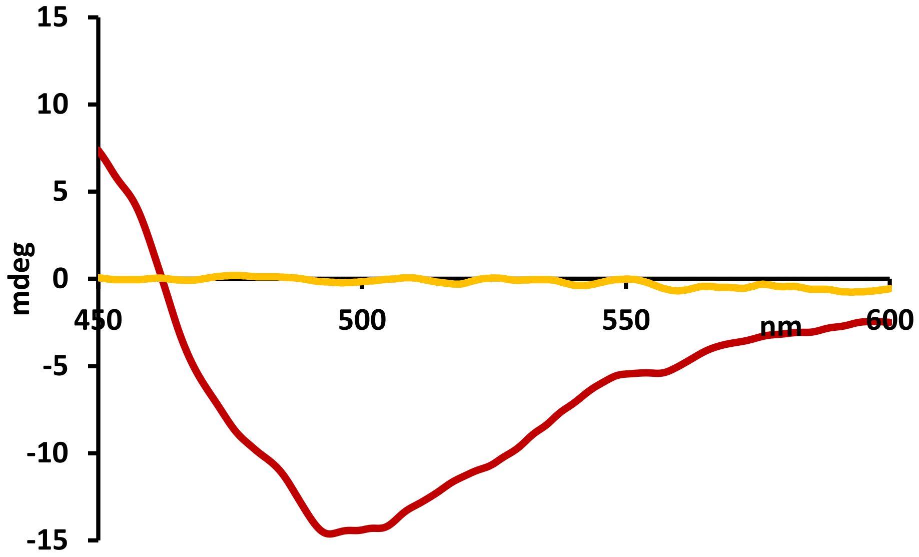

CD measurements were taken at 2.5 mM in MeOH.

Figure S18. CD spectra obtained by applying sensor **4** to dihydroartemisinic acid (**16**, red), vs. **16** only (yellow).

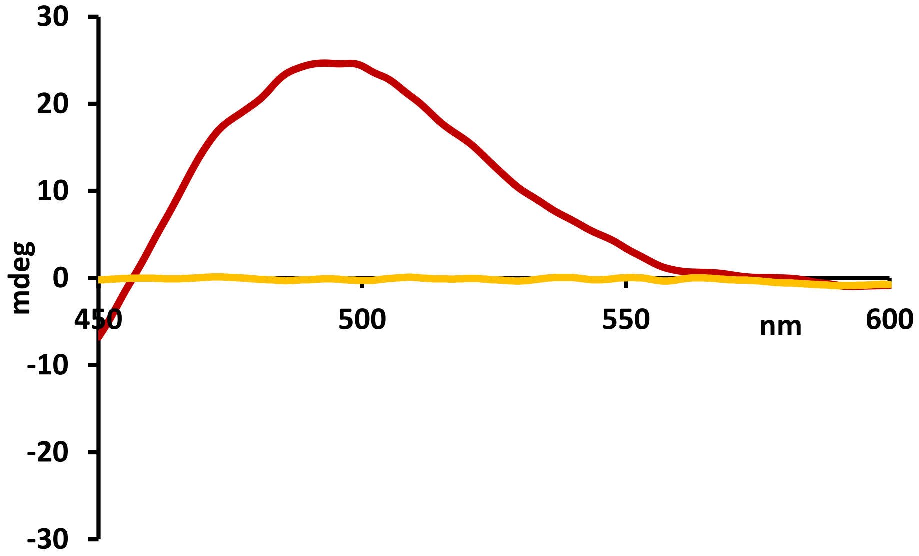

CD measurements were taken at 2.5 mM in MeOH.

Figure S19. CD spectra obtained by applying sensor **4** to (*R*)-**17** (red) vs. (*R*)-**17** only (yellow).

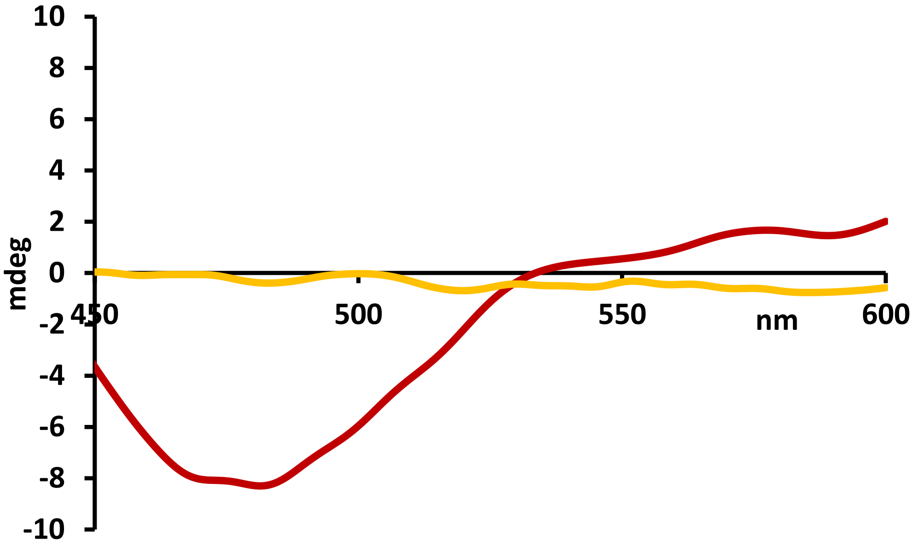

CD measurements were taken at 2.7 mM in MeOH.

Figure S20. CD spectra obtained by applying sensor **4** to (*R*)-**18** (red) and (*S*)-**18** (blue) vs. (*R*)-**18** only (yellow).

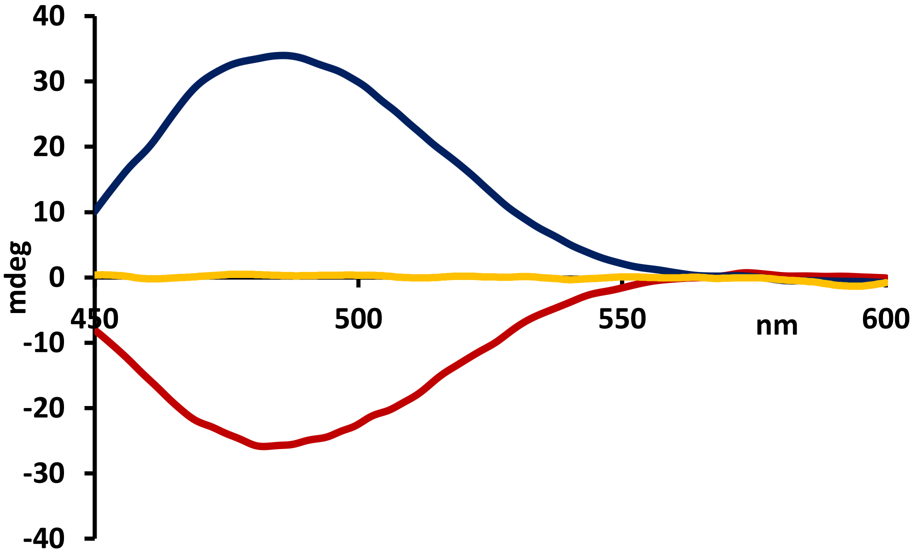

CD measurements were taken at 2.5 mM in MeOH. (*R*)-**18** optical rotation 18.8° (c=1 in dioxane) and (*S*)-**18** optical rotation -21.2° (c=1 in dioxane)

Figure S21. CD spectra obtained by applying sensor **4** to (*R*)-**19** (red) vs. (*R*)-**19** only (yellow).

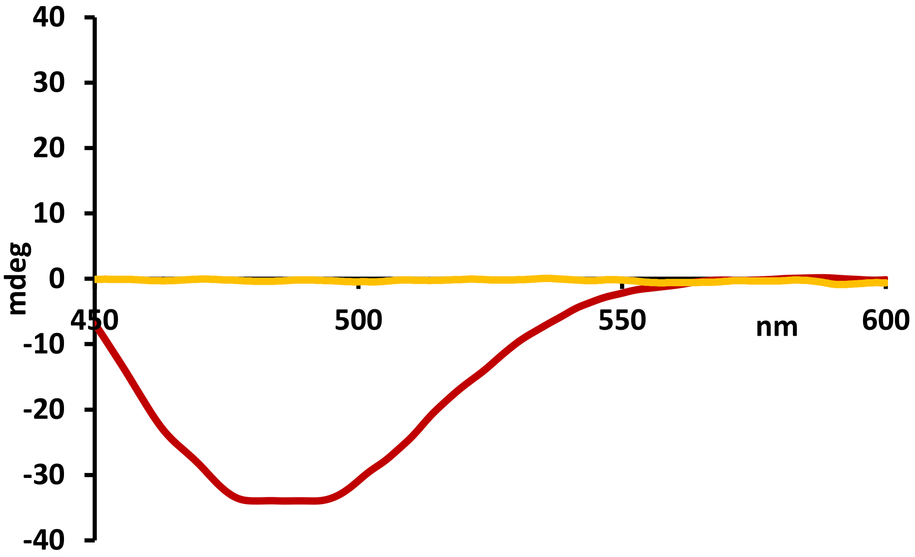

CD measurements were taken at 2.5 mM in MeOH.

Figure S22. CD spectra obtained by applying sensor **4** to (*S*)-**20** (red) vs. (*S*)-**20** only (yellow).

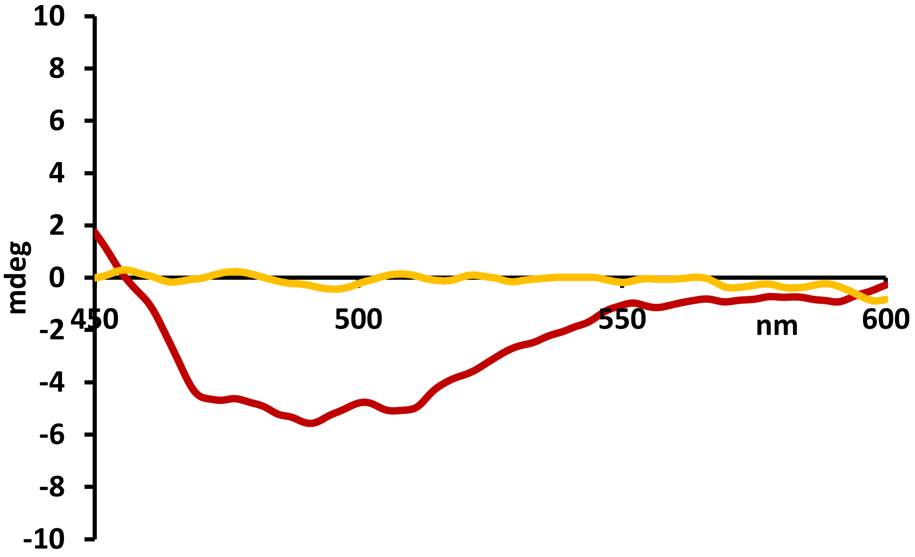

CD measurements were taken at 2.5 mM in MeOH.

Figure S23. CD spectra obtained by applying sensor **4** to (*R*)-**21** (red) vs. (*R*)-**21** only (yellow).

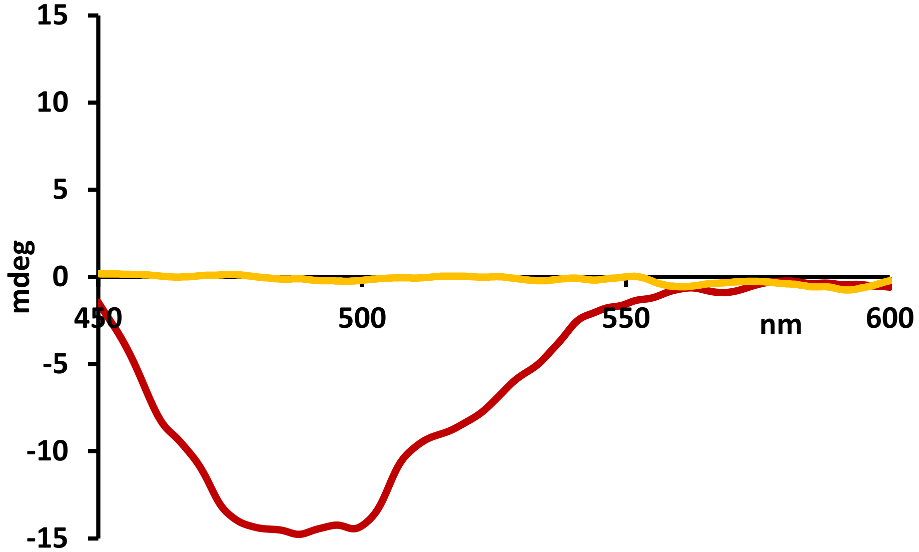

CD measurements were taken at 2.5 mM in MeOH.

Figure S24. CD spectra obtained by applying sensor **4** to (*R*)-**22** (red) and (*S*)-**22** (blue) vs. (*R*)-**22** only (yellow).

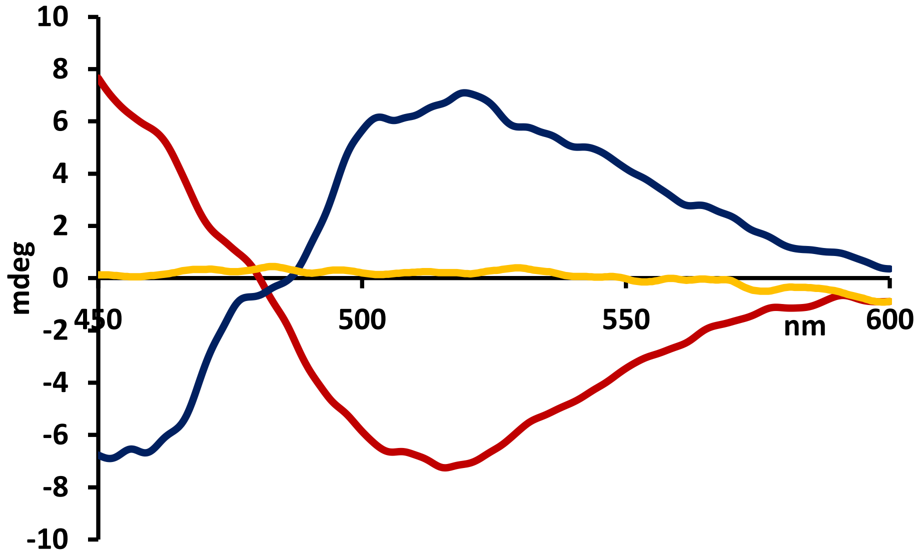

CD measurements were taken at 3.4 mM in MeOH.

Figure S25. CD spectra obtained by applying sensor **4** to (*R*)-**23** (red) and (*S*)-**23** (blue) vs. (*R*)-**23** only (yellow).

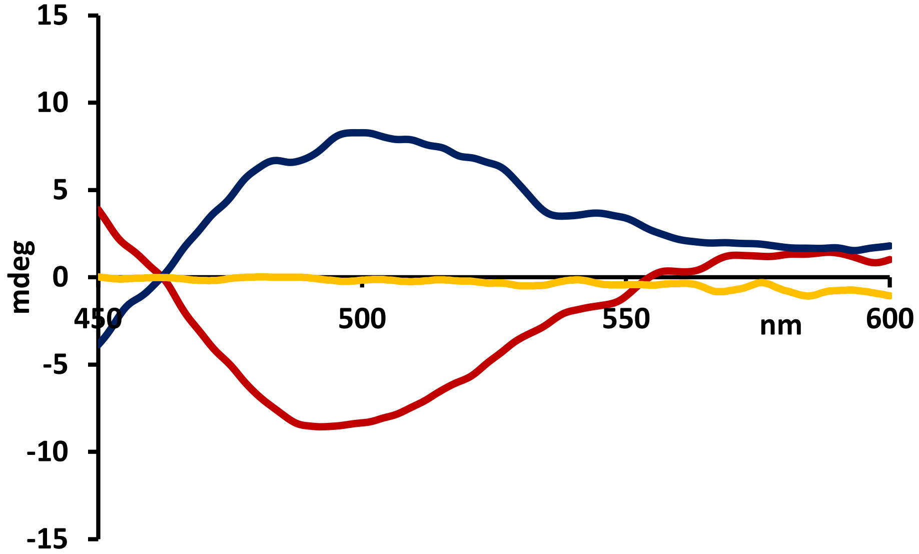

CD measurements were taken at 2.5 mM in MeOH

Figure S26. CD spectra obtained by applying sensor **4** to Tiagabine hydrochloride (**24**, red) vs. **24** only (yellow).

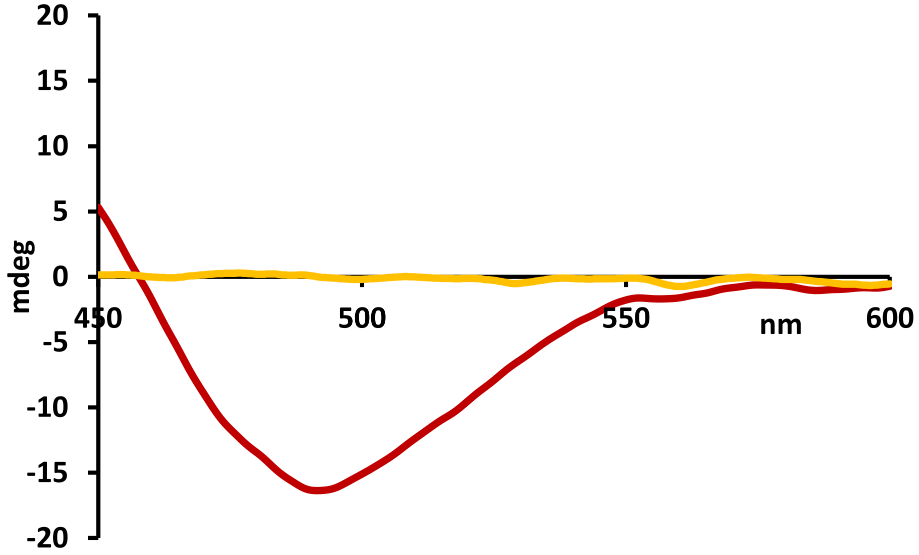

CD measurements were taken at 3.4 mM in MeOH.

Figure S27. CD spectra obtained by applying sensor **4** to (*S*)-**25** (red) and (*R*)-**25** (blue) vs. (*R*)-**25** only (yellow).

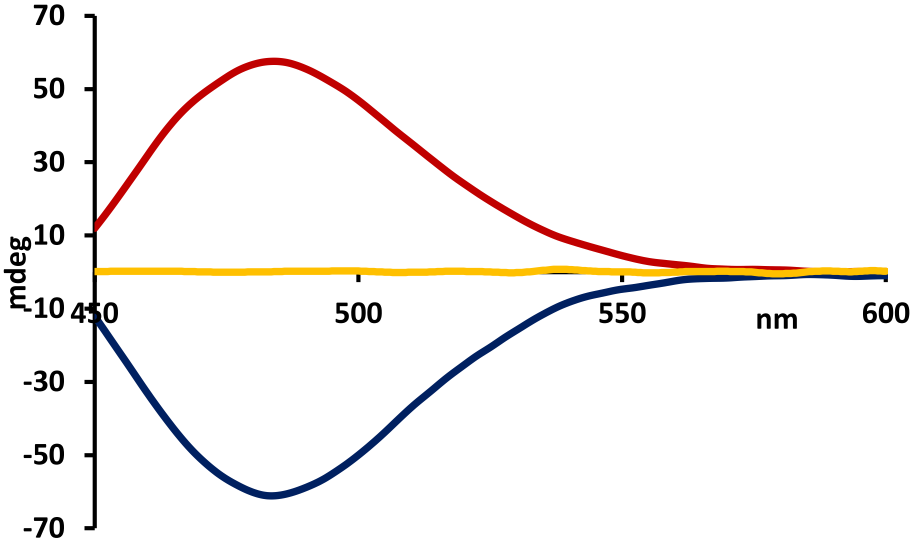

CD measurements were taken at 1.6 mM in MeOH.

Figure S28. CD spectra obtained by applying sensor **4** to (*S*)-**26** (red) and (*R*)-**26** (blue) vs. (*R*)-**26** only (yellow).

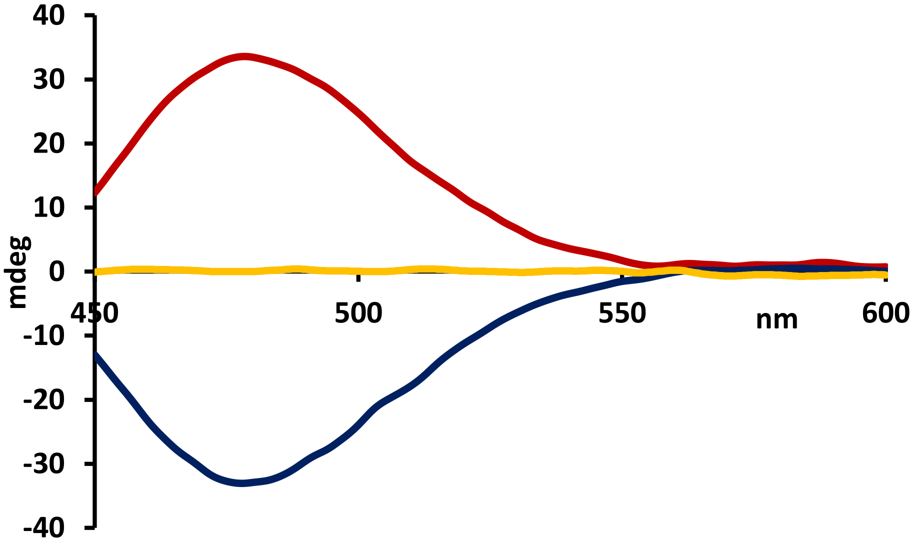

CD measurements were taken at 1.6 mM in MeOH.

Figure S29. CD spectra obtained by applying sensor **4** to (*S*)-**27** (red) and (*R*)-**27** (blue) vs. (*R*)-**27** only (yellow).

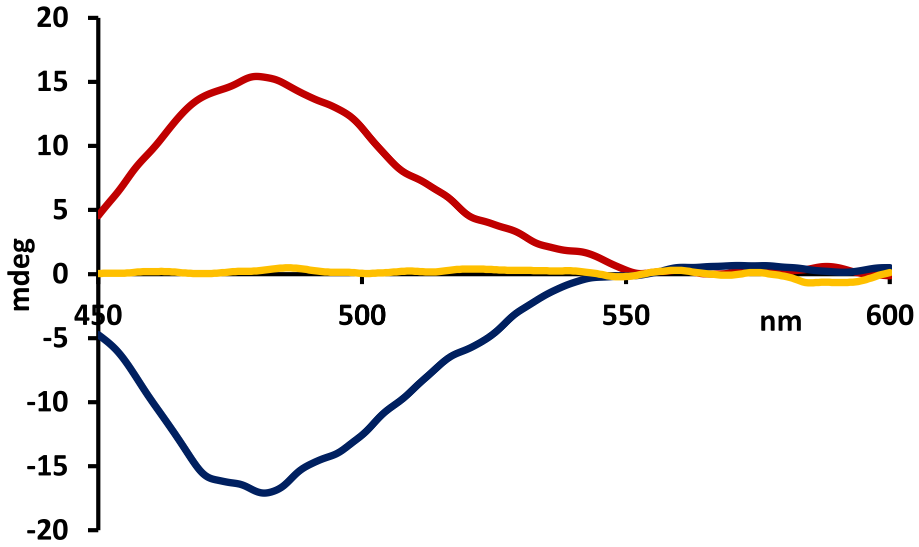

CD measurements were taken at 1.6 mM in MeOH.

Figure S30. CD spectra obtained by applying sensor **4** to (*R*)-**28** (red) and (*S*)-**28** (blue) vs. (*S*)-**28** only (yellow).

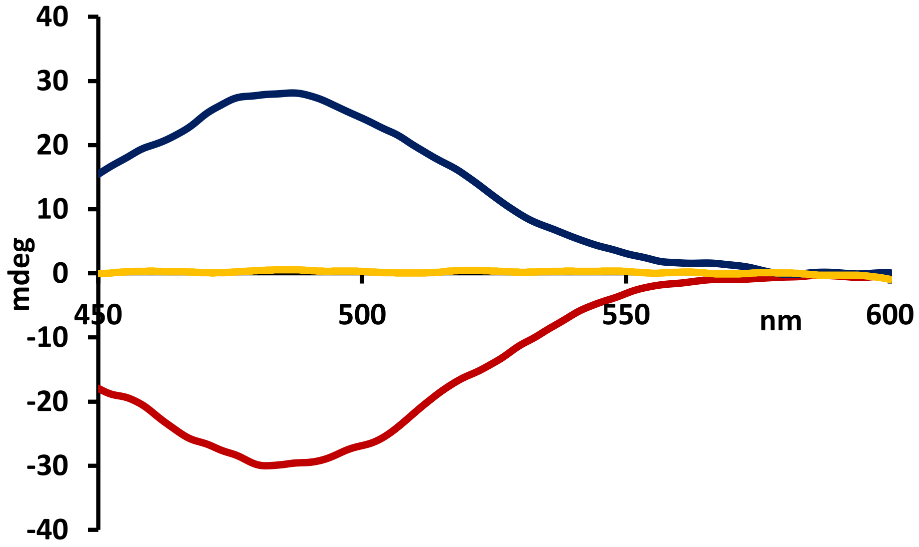

CD measurements were taken at 3.0 mM in MeOH.

Figure S31. CD spectra obtained by applying sensor **4** to (*R*)-**29** (red) and (*S*)-**29** (blue) vs. (*R*)-**29** only (yellow).

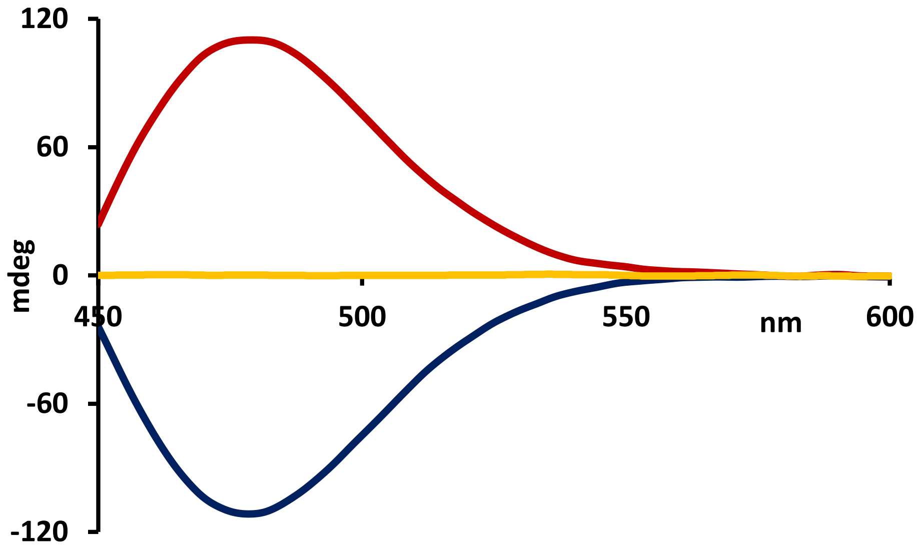

CD measurements were taken at 3.0 mM in MeOH.

Figure S32. CD spectra obtained by applying sensor **4** to (*S*)-**30** (red) vs. (*S*)-**30** only (yellow).

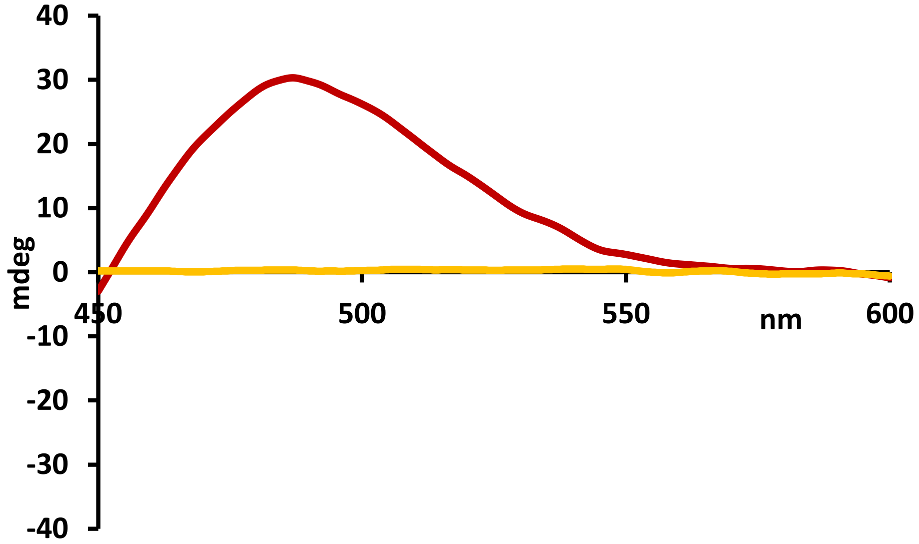

CD measurements were taken at 3.0 mM in MeOH.

Figure S33. CD spectra obtained by applying sensor **4** to (*R*)-2-phenylbutanoic, **31**.

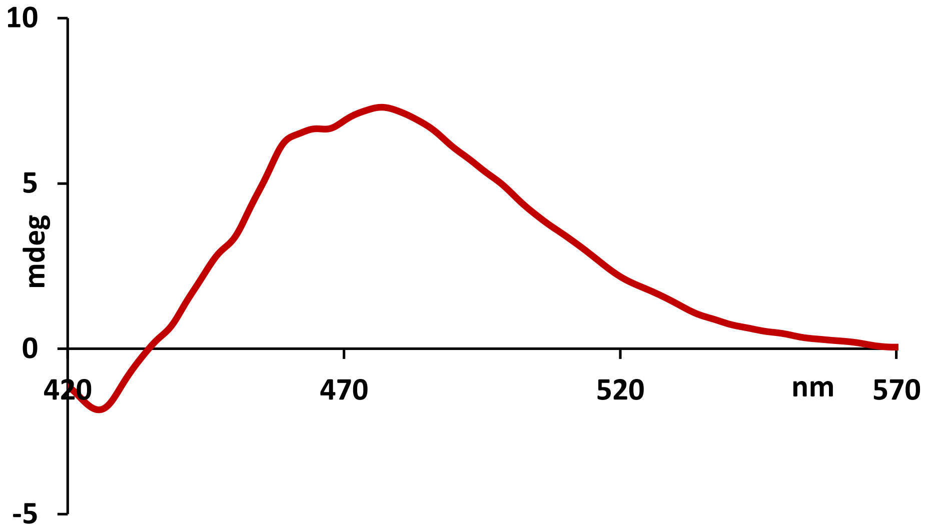

CD measurements were taken at 4.54 mM in MeOH.

Figure S34. CD spectra obtained by applying sensor **4** to (*R*)-3-phenylbutanoic acid, **32**.

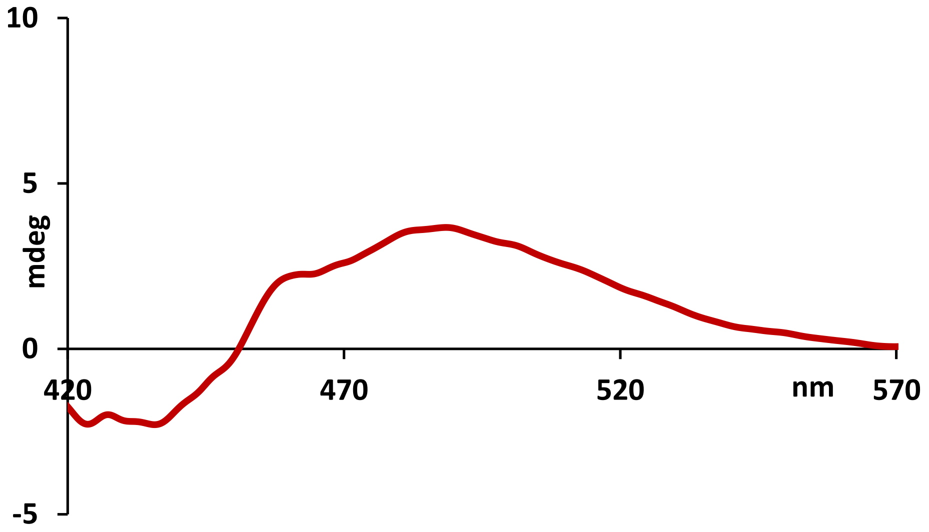

CD measurements were taken at 4.54 mM in MeOH.

Figure S35. CD spectrum of free (*R*)-**8** (250-300 nm).

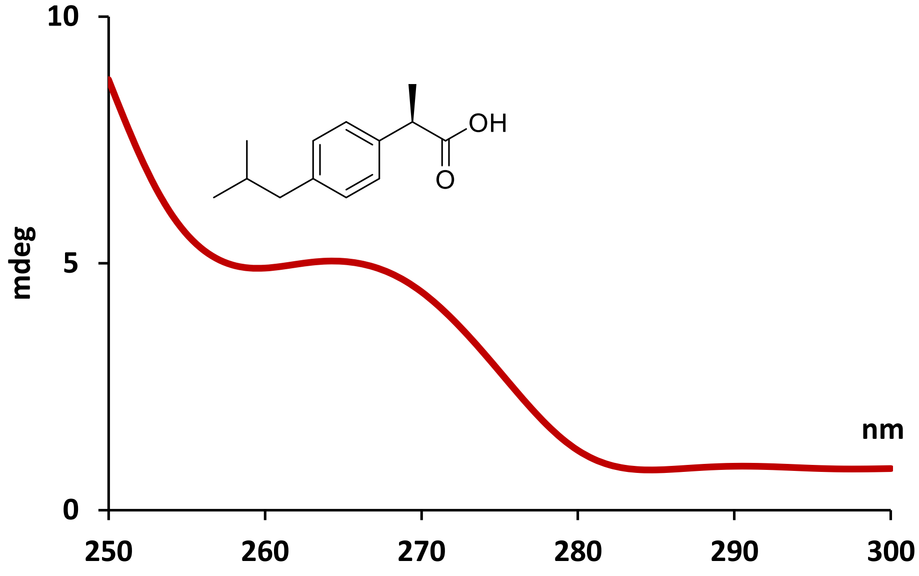

CD measurements were taken at 2.5 mM in CH_2Cl_2.

Figure S36. CD spectrum of free (*R*)-**11** (250-300 nm).

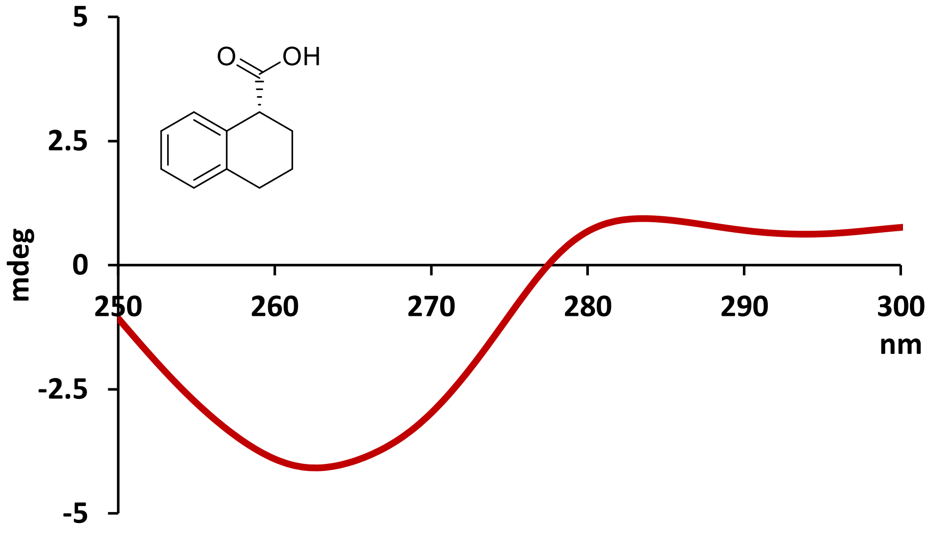

CD measurements were taken at 2.5 mM in CH_2Cl_2.

Figure S37. CD spectrum of free (*S*)-**17** (250-300 nm).

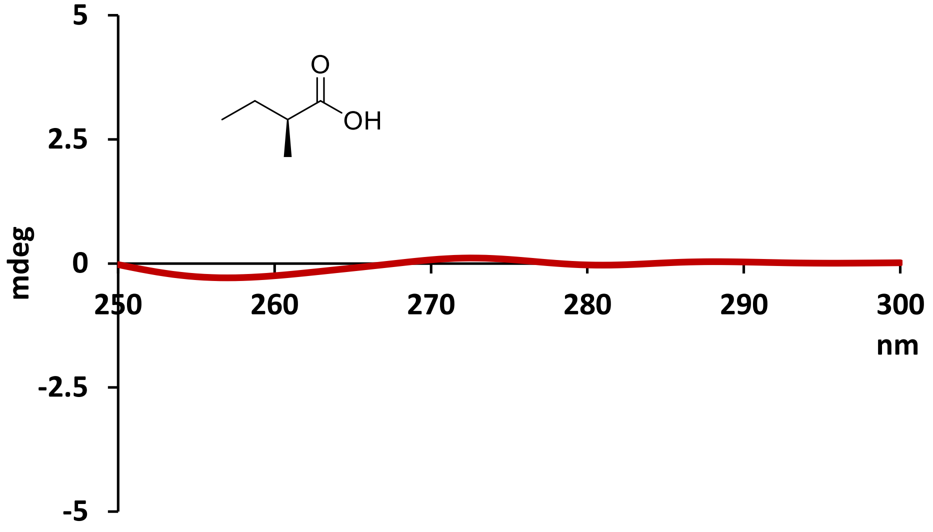

CD measurements were taken at 2.5 mM in CH_2Cl_2.

Figure S38. CD spectrum of free (*R*)-**20** (250-300 nm).

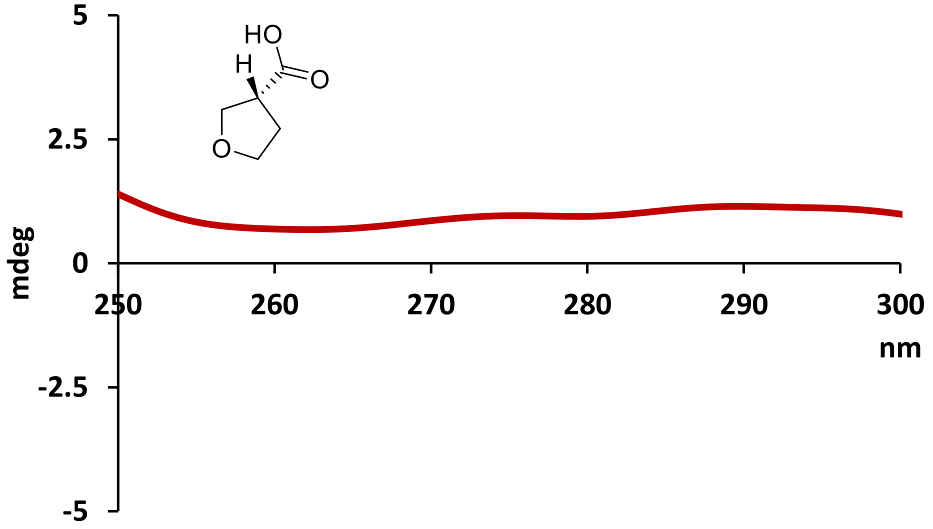

CD measurements were taken at 2.5 mM in CH_2Cl_2.

Figure S39. CD spectrum of free (*R*)-**21** (250-300 nm).

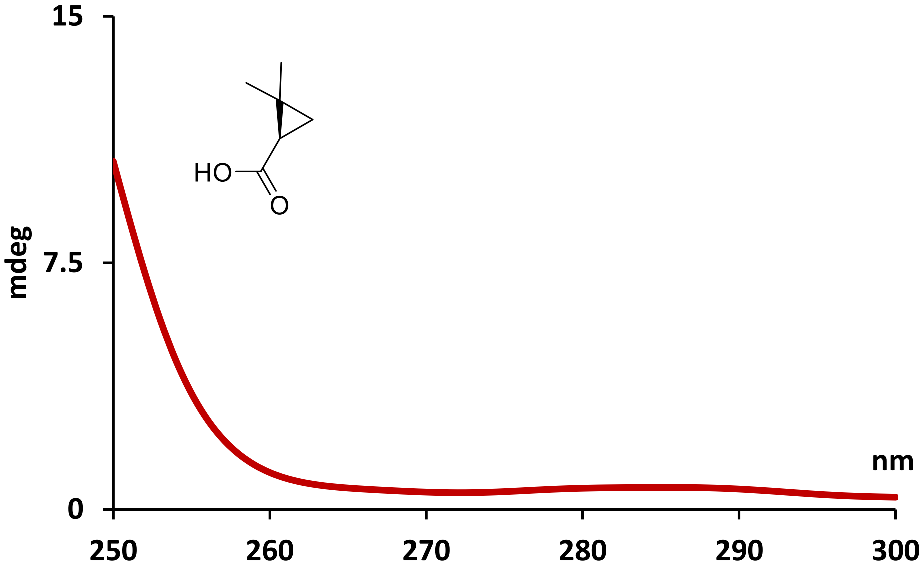

CD measurements were taken at 2.5 mM in CH_2Cl_2.

Figure S40. CD spectrum of free (*R*)-**22** (250-300 nm).

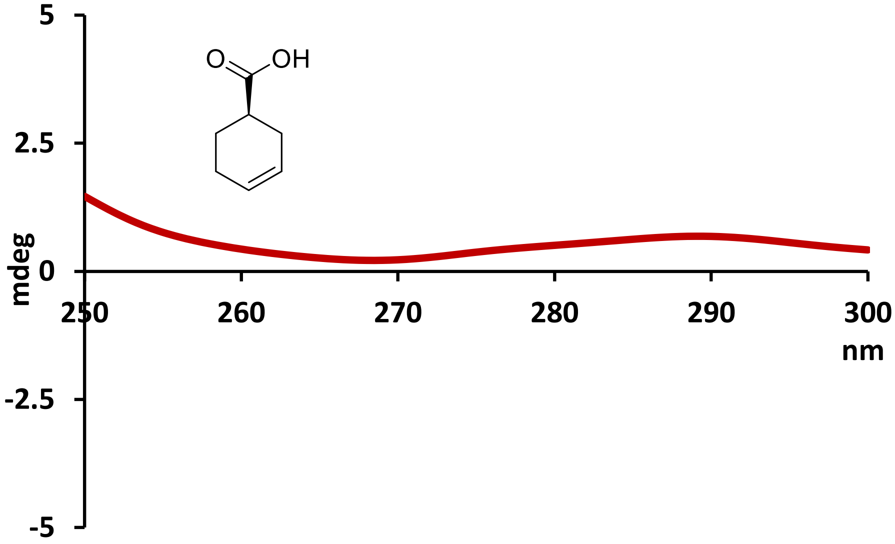

CD measurements were taken at 2.5 mM in CH_2Cl_2.

5. Quantitative carboxylic acid sensing: enantiomeric ratio and total concentration

5.1. Simultaneous concentration and *er* analysis of ibuprofen samples

A sample containing enantioenriched (*S*)-ibuprofen (35.0 mM, 65:35 *er*) was analyzed. To 100.0 μL of the sample were added varying volumes (200.0, 400.0, 600.0, 2200.0 μL) of sensor **4** (12.5 mM in CH_2Cl_2) and varying volumes (10.0, 20.0, 30.0, 110.0 μL) of DIPEA (50.0 mM in CH_2Cl_2). The total reaction volume was adjusted to 2500.0 μL using CH_2Cl_2 and stirred for 15 minutes (first dilution factor X25). CD analysis was performed after diluting 250.0 μL aliquots with 2.0 mL of CH_2Cl_2 (second dilution factor X9).

Figure S41. Plot of CD signals obtained by addition of varying amounts of sensor **4** to an (*S*)-ibuprofen sample (35.0 mM, 65.0:35.0 *er*).

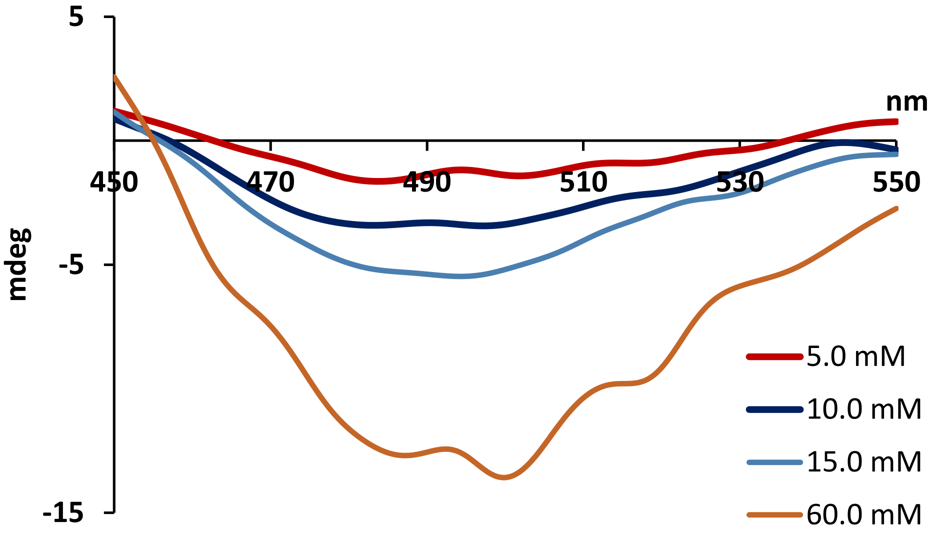

The experimentally obtained CD amplitudes at 490 nm were plotted against concentrations of sensor **4** keeping both dilution factors in mind. Linear regression analysis using the CD amplitudes obtained with **4** in the region of excess of the analyte showed a linear increase (blue line). A horizontal line parallel to the x-axis (slope = 0) representing the range where the CD amplitude is stagnant because the sensor is in excess of the carboxylic acid analyte was obtained (red line). The x-value at the intersection of these two lines was used to determine the original concentration of the carboxylic acid sample (keeping the sample dilution protocol described above in mind) as 32.9 mM. With the concentration of the analyte in hand, the enantiomeric composition was calculated comparing the y-axis value (mdeg) with the of an enantiopure reference. This gave an enantiomeric ratio of 65.5:34.5. The absolute configuration was determined from the sign of the observed CD signal.

Figure S42. Plot CD amplitudes at 490 nm obtained with varying amounts of the sensor and (*S*)-ibuprofen (35.0 mM, 65.0:35.0 *er*).

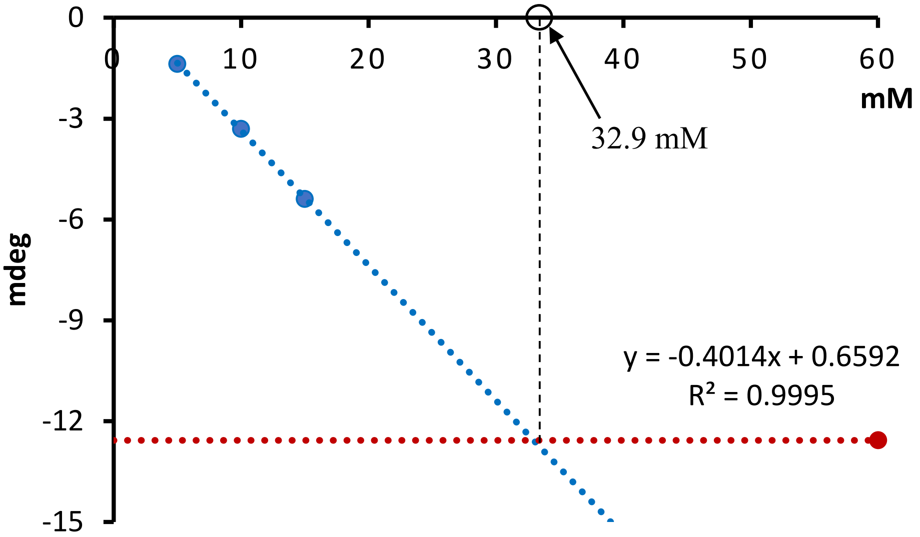

A sample containing enantioenriched (*S*)-ibuprofen (40.0 mM, 70:30 *er*) was analyzed. To 100.0 µL of the sample were added varying volumes (200.0, 400.0, 600.0, 2200.0 µL) of sensor **4** (12.5 mM in CH_2Cl_2) and varying volumes (10.0, 20.0, 30.0, 110.0 µL) of DIPEA (50.0 mM in CH_2Cl_2). The total reaction volume was adjusted to 2500.0 µL using CH_2Cl_2 and stirred for 15 minutes. CD analysis was performed after diluting 250.0 µL aliquot with 2.0 mL of CH_2Cl_2. The concentration and enantiomeric ratio were determined as 40.3 mM and 71.0:29.0 *er* using the protocol mentioned above.

Figure S43. Plot of CD signals obtained by addition of varying amounts of sensor **4** to an (*S*)-ibuprofen sample (40.0 mM, 70.0:30.0 *er*).

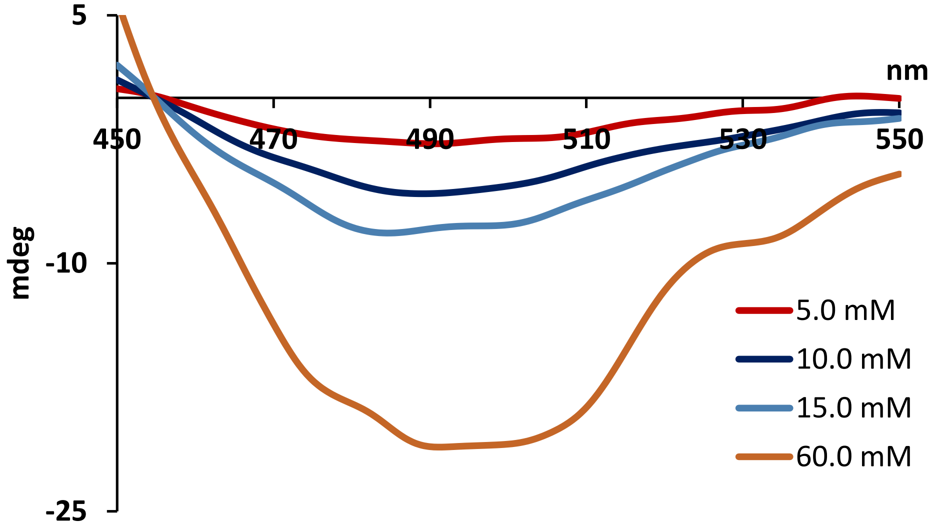

Figure S44. Plot CD amplitudes at 490 nm obtained with varying amounts of the sensor and (*S*)-ibuprofen (40.0 mM, 70.0:30.0 *er*).

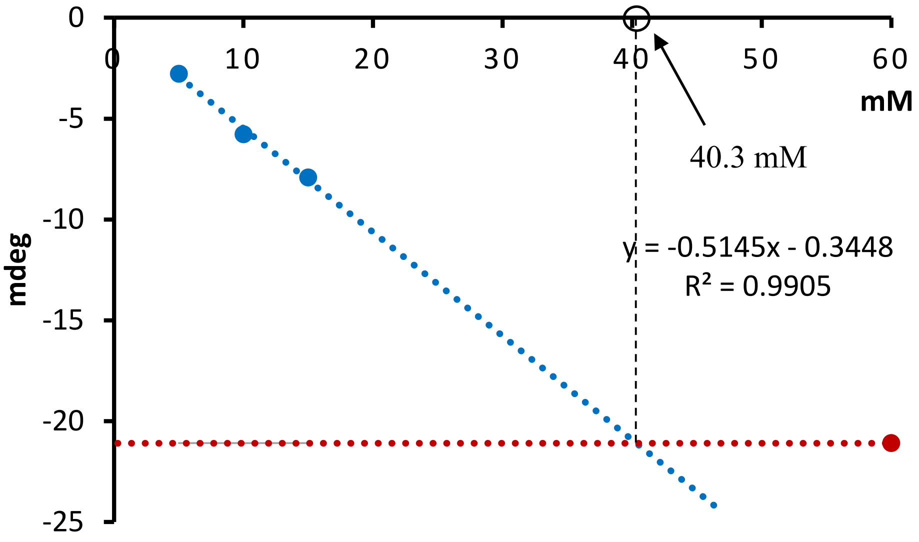

A sample containing enantioenriched (*S*)-ibuprofen (45.0 mM, 60:40 *er*) was analyzed. To 100.0 μL of the sample were added varying volumes (200.0, 400.0, 600.0, 2200.0 μL) of sensor **4** (12.5 mM in CH_2Cl_2) and varying volumes (10.0, 20.0, 30.0, 110.0 μL) of DIPEA (50.0 mM in CH_2Cl_2). The total reaction volume was adjusted to 2500.0 μL using CH_2Cl_2 and stirred for 15 minutes. CD analysis was performed after diluting 250.0 μL aliquot with 2.0 mL of CH_2Cl_2. The concentration and enantiomeric ratio were determined as 43.7 mM and 67.0:33.0 *er* using the protocol mentioned above.

Figure S45. Plot of CD signals obtained by addition of varying amounts of sensor **4** to an (*S*)-ibuprofen sample (45.0 mM, 60.0:40.0 *er*).

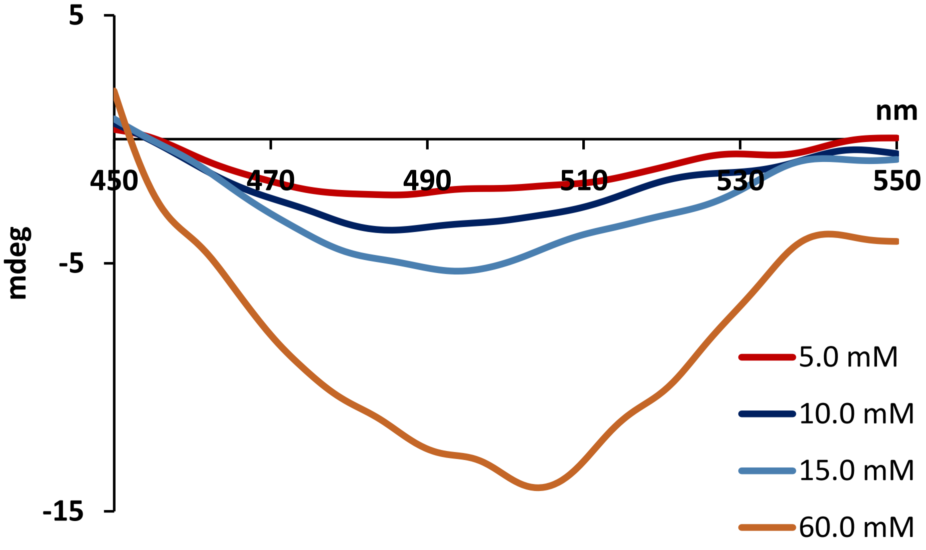

Figure S46. Plot CD amplitudes at 490 nm obtained with varying amounts of the sensor and (*S*)-ibuprofen (45.0 mM, 60.0:40.0 *er*).

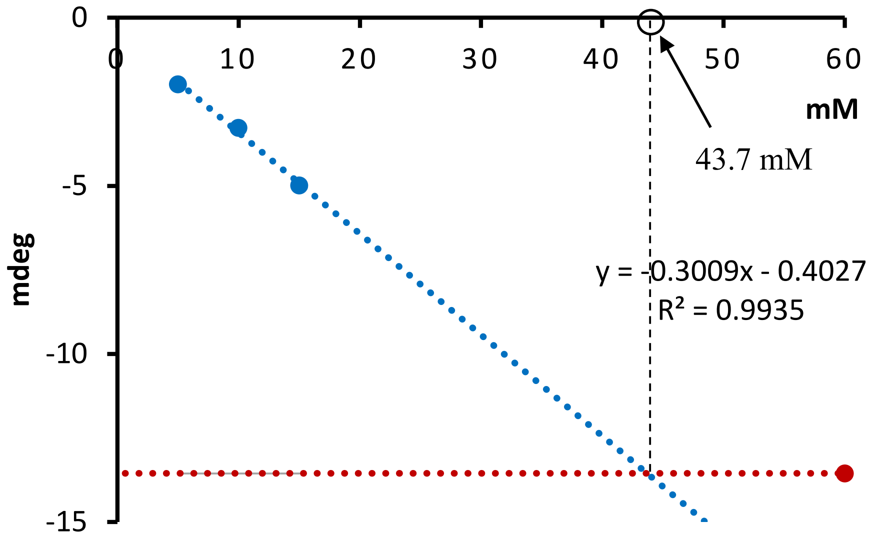

A sample containing enantioenriched (*S*)-ibuprofen (30.0 mM, 95:5 *er*) was analyzed. To 100.0 μL of the sample were added varying volumes (200.0, 400.0, 600.0, 2200.0 μL) of sensor **4** (12.5 mM in CH_2Cl_2) and varying volumes (10.0, 20.0, 30.0, 110.0 μL) of DIPEA (50.0 mM in CH_2Cl_2). The total reaction volume was adjusted to 2500.0 μL using CH_2Cl_2 and stirred for 15 minutes. CD analysis was performed after diluting 250.0 μL aliquot with 2.0 mL of CH_2Cl_2. The concentration and enantiomeric ratio were determined as 33.0 mM and 90.5:9.5 *er* using the protocol mentioned above.

Figure S47. Plot of CD signals obtained by addition of varying amounts of sensor **4** to an (*S*)-ibuprofen sample (30.0 mM, 95.0:5.0 *er*).

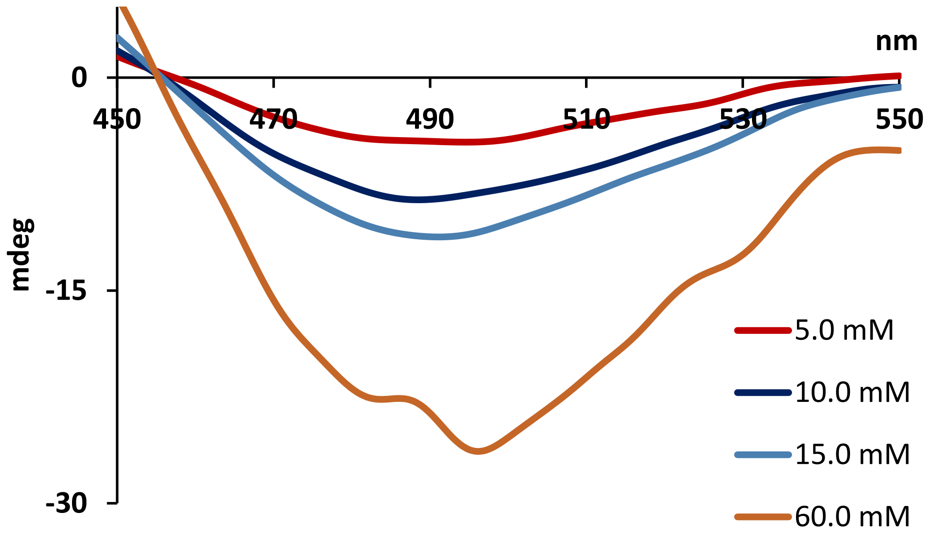

Figure S48. Plot CD amplitudes at 490 nm obtained with varying amounts of the sensor and (*S*)-ibuprofen (30.0 mM, 95.0:5.0 *er*).

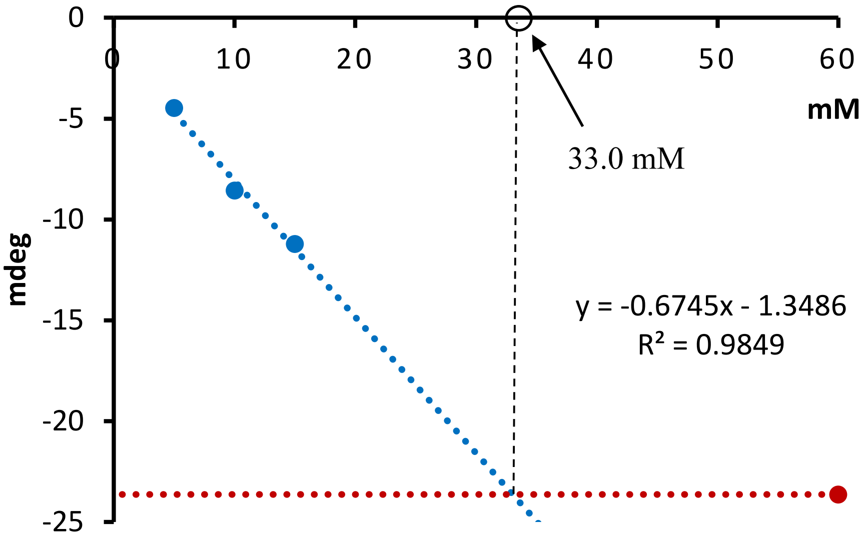

A sample containing enantioenriched (*S*)-ibuprofen (38.0 mM, 80:20 *er*) was analyzed. To 100.0 µL of the sample were added varying volumes (200.0, 400.0, 600.0, 2200.0 µL) of sensor **4** (12.5 mM in CH_2Cl_2) and varying volumes (10.0, 20.0, 30.0, 110.0 µL) of DIPEA (50.0 mM in CH_2Cl_2). The total reaction volume was adjusted to 2500.0 µL using CH_2Cl_2 and stirred for 15 minutes. CD analysis was performed after diluting 250.0 µL aliquot with 2.0 mL of CH_2Cl_2. The concentration and enantiomeric ratio were determined as 33.0 mM and 84.0:16.0 *er* using the protocol mentioned above.

Figure S49. Plot of CD signals obtained by addition of varying amounts of sensor **4** to an (*S*)-ibuprofen sample (38.0 mM, 80.0:20.0 *er*).

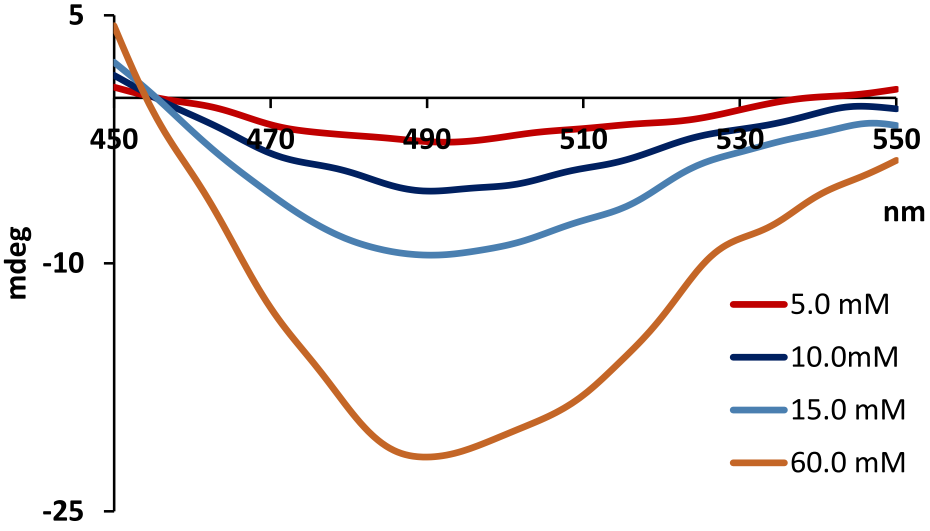

Figure S50. Plot CD amplitudes at 490 nm obtained with varying amounts of the sensor and (*S*)-ibuprofen (38.0 mM, 80.0:20.0 *er*).

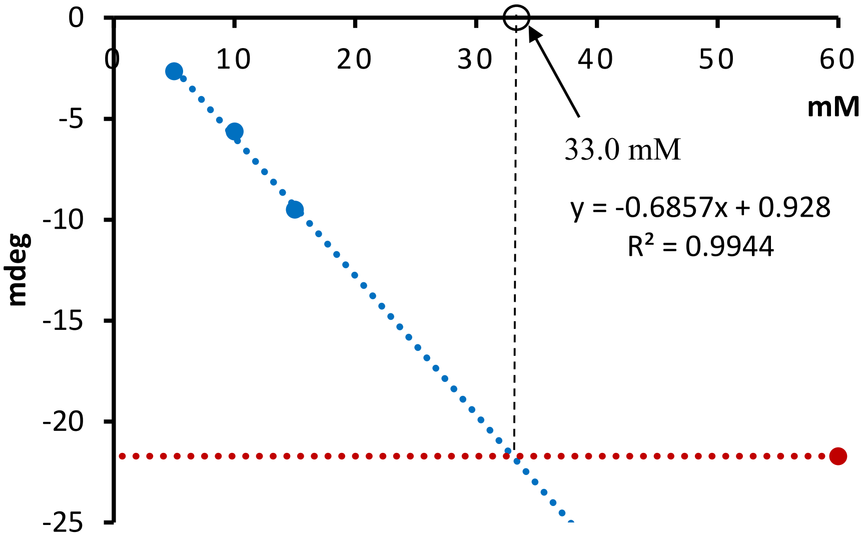

5.2. Simultaneous concentration and *er* analysis of 1,2,3,4-tetrahydronaphthalene-1-carboxylic acid samples

A sample containing enantioenriched (*R*)- 1,2,3,4-tetrahydronaphthalene-1-carboxylic acid (35.0 mM, 80:20 *er*) was analyzed. To 100.0 μL of the sample were added varying volumes (200.0, 400.0, 600.0, 2200.0 μL) of sensor **4** (12.5 mM in CH_2Cl_2) and varying volumes (10.0, 20.0, 30.0, 110.0 μL) of DIPEA (50.0 mM in CH_2Cl_2). The total reaction volume was adjusted to 2500.0 μL using CH_2Cl_2 and stirred for 15 minutes (first dilution factor X25). CD analysis was performed after diluting 250.0 μL aliquot with 2.0 mL of CH_2Cl_2 (second dilution factor X9). The concentration and enantiomeric ratio were determined as 36.7 mM and 79.0:21.0 *er* using the protocol mentioned above.

Figure S51. Plot of CD signals obtained by addition of varying amounts of sensor **4** to an (*R*)-1,2,3,4-tetrahydronaphthalene-1-carboxylic acid sample (35.0 mM, 80.0:20.0 *er*).

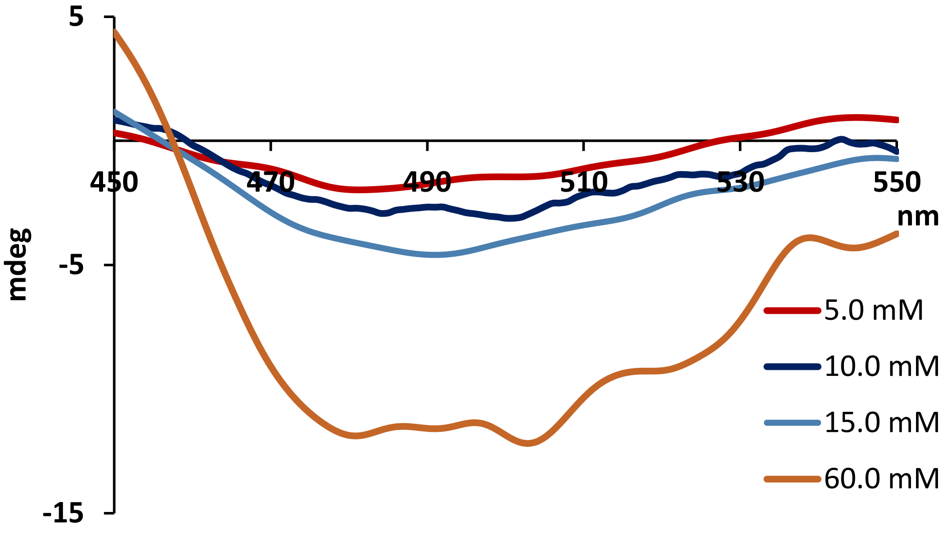

Figure S52. Plot CD amplitudes at 490 nm obtained with varying amounts of the sensor and (*R*)-1,2,3,4-tetrahydronaphthalene-1-carboxylic acid (35.0 mM, 80.0:20.0 *er*).

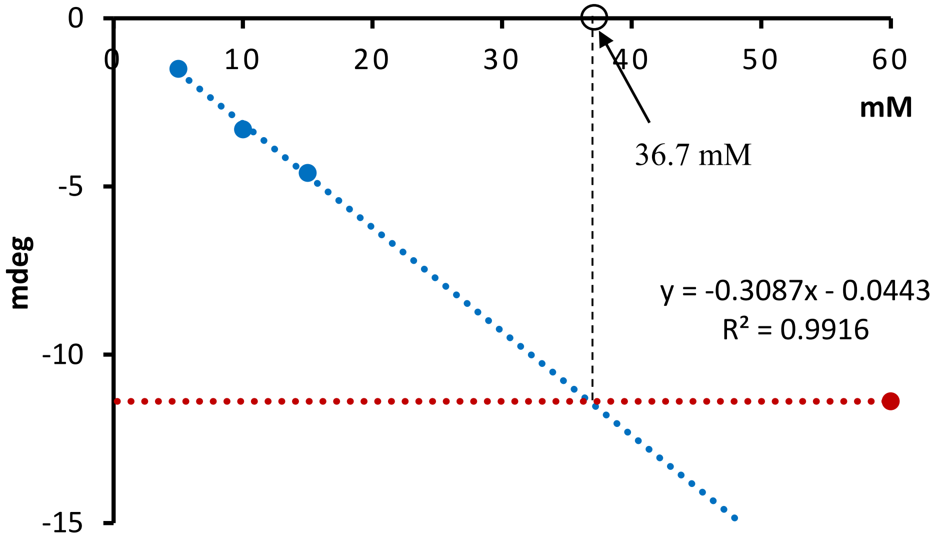

A sample containing enantioenriched (*S*)- 1,2,3,4-tetrahydronaphthalene-1-carboxylic acid (25.0 mM, 65:35 *er*) was analyzed. To 100.0 μL of the sample were added varying volumes (200.0, 400.0, 600.0, 2200.0 μL) of sensor **4** (12.5 mM in CH_2Cl_2) and varying volumes (10.0, 20.0, 30.0, 110.0 μL) of DIPEA (50.0 mM in CH_2Cl_2). The total reaction volume was adjusted to 2500.0 μL using CH_2Cl_2 and stirred for 15 minutes. CD analysis was performed after diluting 250.0 μL aliquot with 2.0 mL of CH_2Cl_2. The concentration and enantiomeric ratio were determined as 20.0 mM and 72.5:28.5 *er* using the protocol mentioned above.

Figures S53. Plot of CD signals obtained by addition of varying amounts of sensor **4** to an (*S*)-1,2,3,4-tetrahydronaphthalene-1-carboxylic acid sample (25.0 mM, 65.0:35.0 *er*).

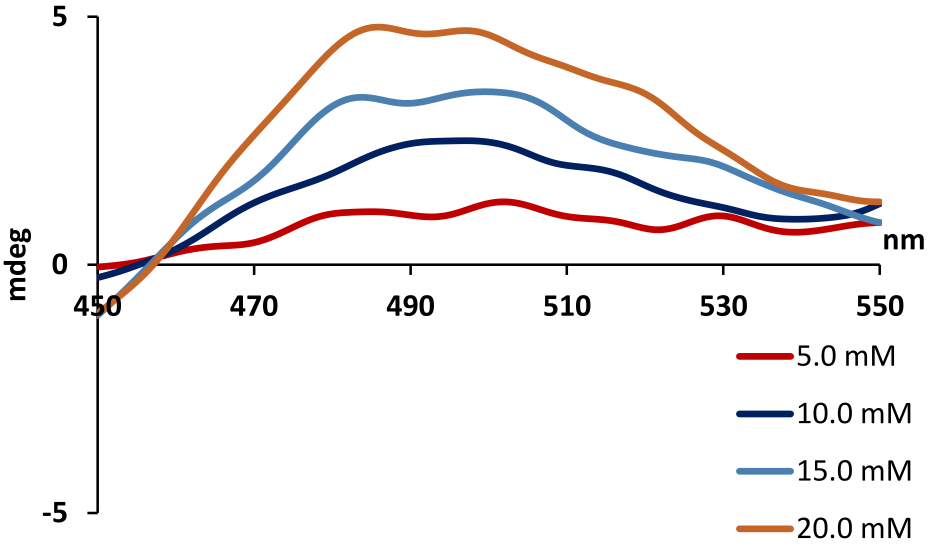

Figure S54. Plot CD amplitudes at 490 nm obtained with varying amounts of the sensor and (*S*)-1,2,3,4-tetrahydronaphthalene-1-carboxylic acid (25.0 mM, 65.0:35.0 *er*).

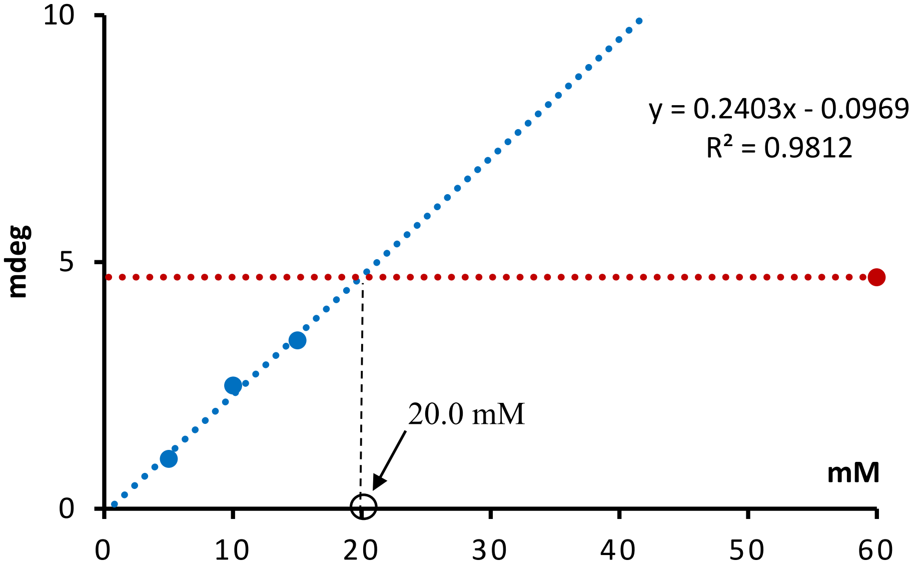

A sample containing enantioenriched (*S*)- 1,2,3,4-tetrahydronaphthalene-1-carboxylic acid (30.0 mM, 70:30 *er*) was analyzed. To 100.0 µL of the sample were added varying volumes (200.0, 400.0, 600.0, 2200.0 µL) of sensor **4** (12.5 mM in CH_2Cl_2) and varying volumes (10.0, 20.0, 30.0, 110.0 µL) of DIPEA (50.0 mM in CH_2Cl_2). The total reaction volume was adjusted to 2500.0 µL using CH_2Cl_2 and stirred for 15 minutes. CD analysis was performed after diluting 250.0 µL aliquot with 2.0 mL of CH_2Cl_2. The concentration and enantiomeric ratio were determined as 32.0 mM and 75.0:25.0 *er* using the protocol mentioned above.

Figure S55. Plot of CD signals obtained by addition of varying amounts of sensor **4** to an (*S*)-1,2,3,4-tetrahydronaphthalene-1-carboxylic acid sample (30.0 mM, 70.0:30.0 *er*).

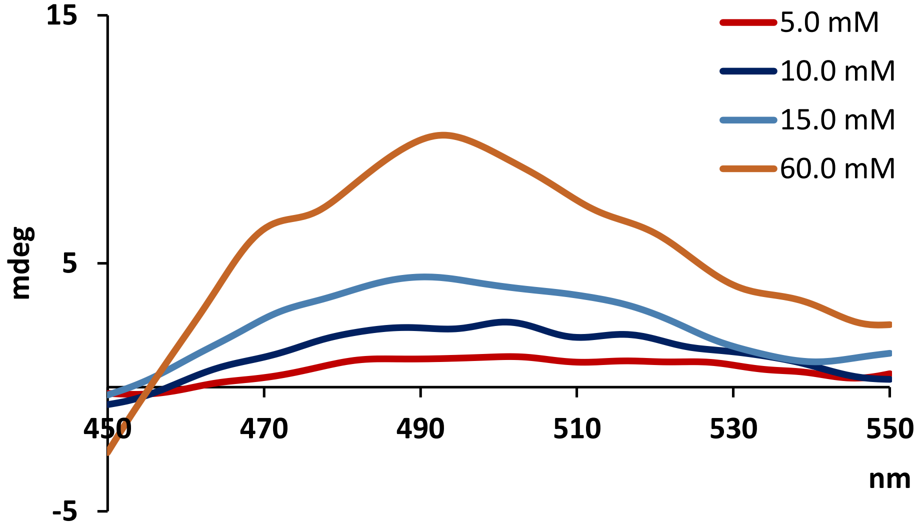

Figure S56. Plot CD amplitudes at 490 nm obtained with varying amounts of the sensor and (*S*)-1,2,3,4-tetrahydronaphthalene-1-carboxylic acid (30.0 mM, 70.0:30.0 *er*).

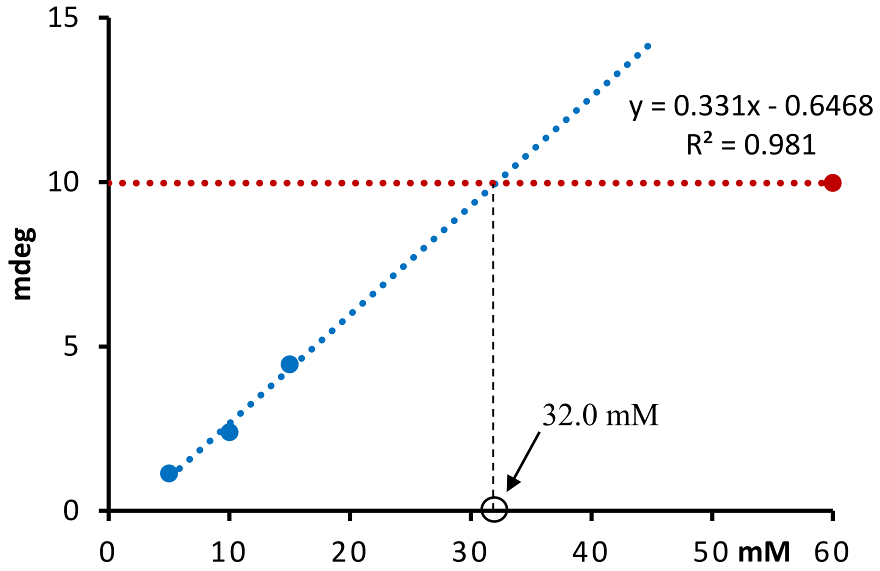

A sample containing enantioenriched (*R*)- 1,2,3,4-tetrahydronaphthalene-1-carboxylic acid (28.0 mM, 10:90 *er*) was analyzed. To 100.0 µL of the sample were added varying volumes (200.0, 400.0, 600.0, 2200.0 µL) of sensor **4** (12.5 mM in CH_2Cl_2) and varying volumes (10.0, 20.0, 30.0, 110.0 µL) of DIPEA (50.0 mM in CH_2Cl_2). The total reaction volume was adjusted to 2500.0 µL using CH_2Cl_2 and stirred for 15 minutes. CD analysis was performed after diluting 250.0 µL aliquot with 2.0 mL of CH_2Cl_2.The concentration and enantiomeric ratio were determined as 26.0 mM and 5.5:94.5 *er* using the protocol mentioned above.

Figure S57. Plot of CD signals obtained by addition of varying amounts of sensor **4** to an (*R*)-1,2,3,4-tetrahydronaphthalene-1-carboxylic acid sample (28.0 mM, 10.0:90.0 *er*).

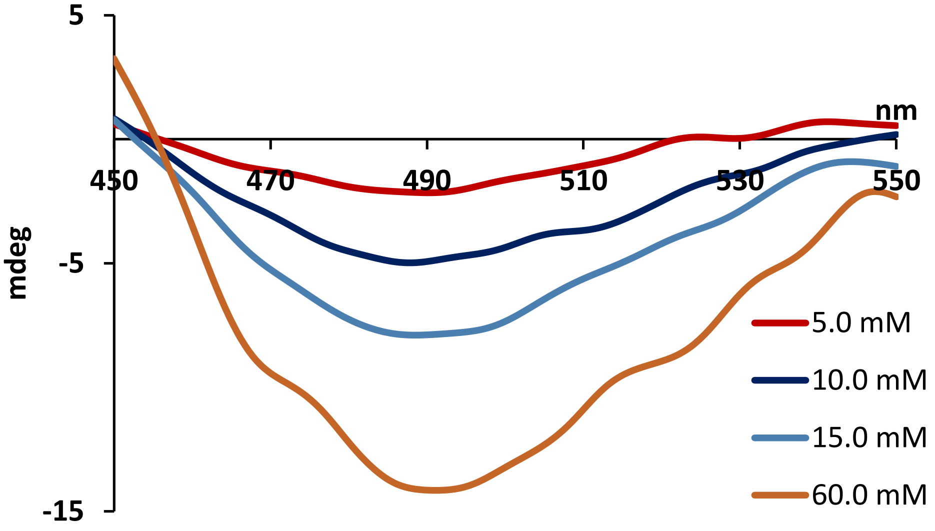

Figure S58. Plot CD amplitudes at 490 nm obtained with varying amounts of the sensor and (*R*)-1,2,3,4-tetrahydronaphthalene-1-carboxylic acid (28.0 mM, 10.0:90.0 *er*).

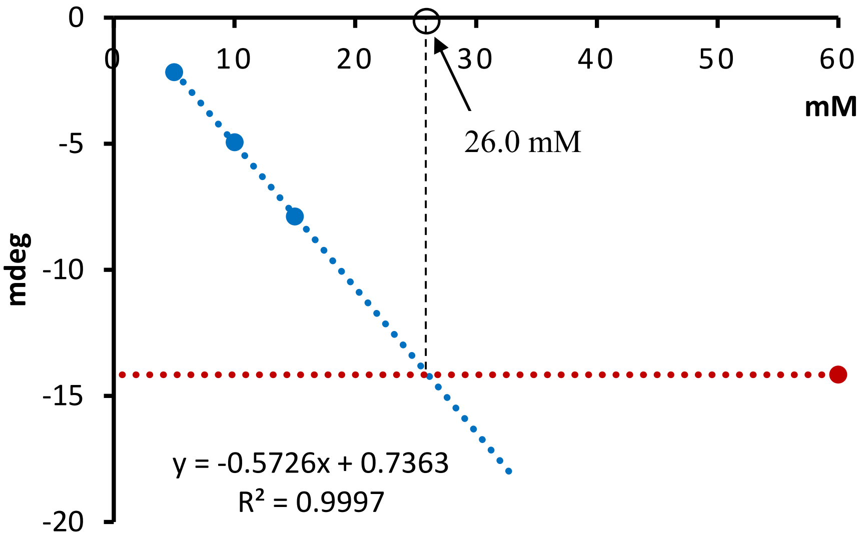

A sample containing enantioenriched (*R*)- 1,2,3,4-tetrahydronaphthalene-1-carboxylic acid (25.0 mM, 25:75 *er*) was analyzed. To 100.0 μL of the sample were added varying volumes (200.0, 400.0, 600.0, 2200.0 μL) of sensor **4** (12.5 mM in CH_2Cl_2) and varying volumes (10.0, 20.0, 30.0, 110.0 μL) of DIPEA (50.0 mM in CH_2Cl_2). The total reaction volume was adjusted to 2500.0 μL using CH_2Cl_2 and stirred for 15 minutes. CD analysis was performed after diluting 250.0 μL aliquot with 2.0 mL of CH_2Cl_2. The concentration and enantiomeric ratio were determined as 28.9 mM and 22.5:77.5 *er* using the protocol mentioned above.

Figure S59. Plot of CD signals obtained by addition of varying amounts of sensor **4** to an (*R*)-1,2,3,4-tetrahydronaphthalene-1-carboxylic acid sample (25.0 mM, 25.0:75.0 *er*).

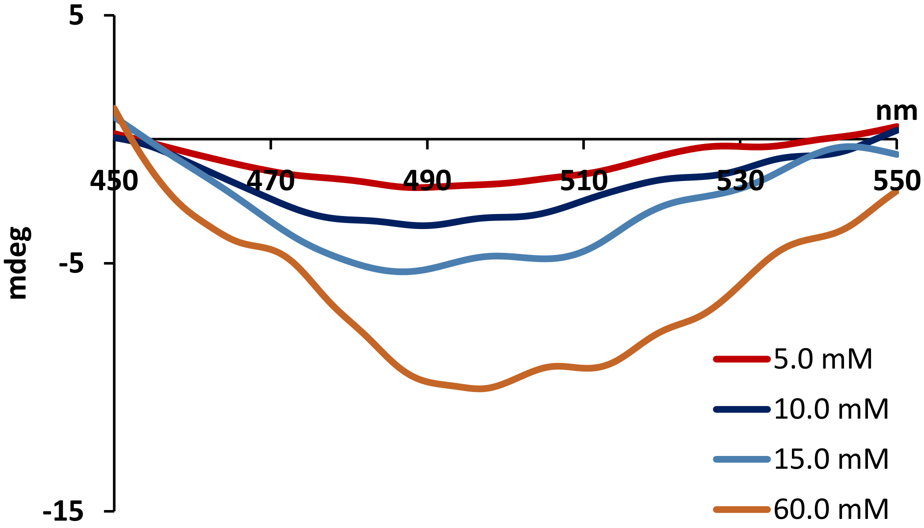

Figure S60. Plot CD amplitudes at 490 nm obtained with varying amounts of the sensor and (*R*)-1,2,3,4-tetrahydronaphthalene-1-carboxylic acid (25.0 mM, 25.0:75.0 *er*).

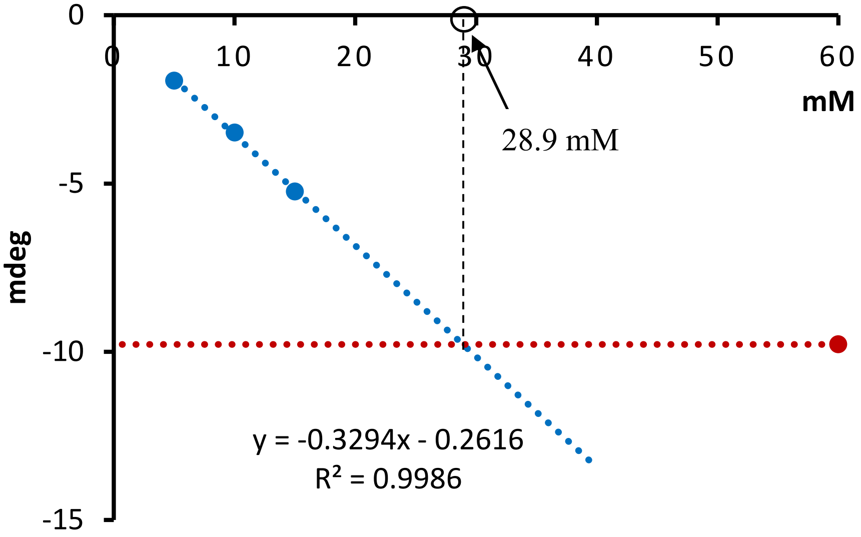

Table S1. Analysis of absolute configuration, *er*, and concentration of Ibuprofen and 1,2,3,4-tetrahydronaphthalene-1-carboxylic acid with sensor **4** by CD sensing.

Sample	Sample Composition			CD sensing results		
	Abs. Config.	Conc (mM)	*er*	Abs. Config.	Conc (mM)	*er*
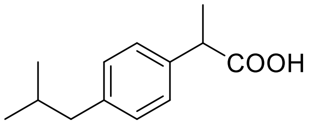	*S*	35.0	65.0:35.0	*S*	32.9	65.5:34.5
	S	40.0	70.0:30.0	*S*	40.3	71.0:29.0
	S	45.0	60.0:40.0	*S*	43.7	67.0:33.0
	S	30.0	95.0:5.0	*S*	33.0	90.5:9.5
	S	38.0	80.0:20.0	*S*	33.0	84.0:16.0
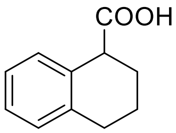	*R*	35.0	20.0:80.0	*R*	36.7	21.0:79.0
	S	25.0	65.0:35.0	*S*	20.0	72.5:27.5
	S	30.0	70.0:30.0	*S*	32.0	75.0:25.0
	R	28.0	10.0:90.0	*R*	26.0	5.5:94.5
	R	25.0	25.0:75.0	*R*	28.9	22.5:77.5

To determine the error margins of the concentration and *er* determinations originating from the maximum CD induction deviations observed, the mdeg values were varied by 0.5 mdeg for all four measurements of Sample #1. An averaged (maximum) deviation of 2.08 mM, 6.3% (3.8 mM, 11.6%) was calculated by this analysis. The *er* value on the other hand changed only slightly. The averaged (maximum) *er* deviation calculated was 66.1:33.9 which corresponds to 2.7% (67.0:33.0 or 6.5%).

Figure S61. Original data of Sample #1

Chiroptical sensing results: 32.9 mM, 65.5:34.5 *er*

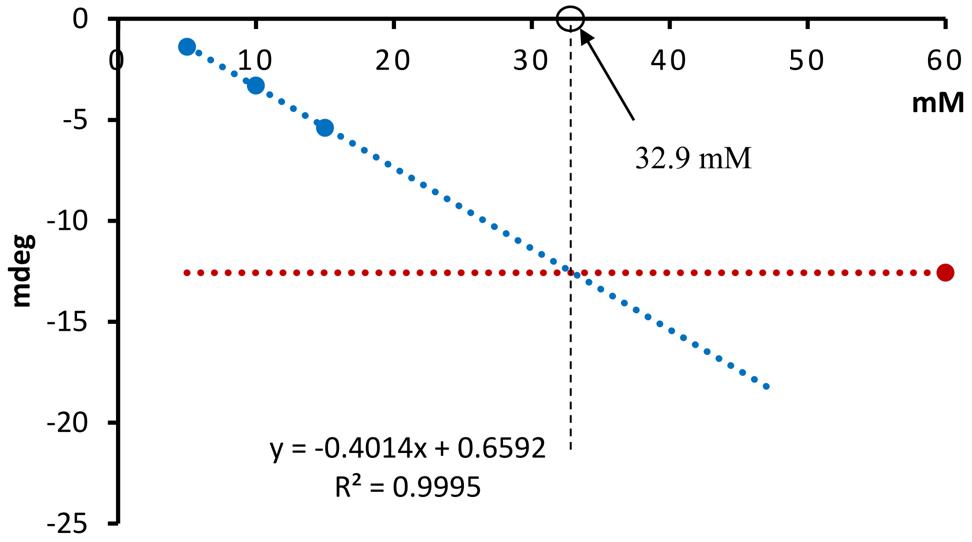

Figure S62. Variation of the 1st measurement

The ICD (mdeg) value of the 1st measurement was decreased by -0.5 mdeg

Chiroptical sensing results: 35.7 mM, 66.5:33.5 *er*

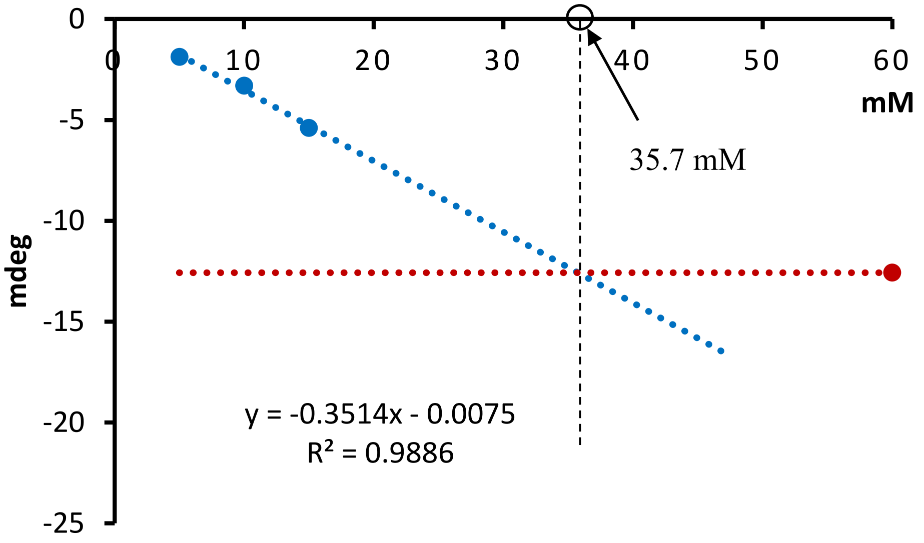

Figure S63. Variation of the 2nd measurement

The ICD (mdeg) value of the 2nd measurement was increased by +0.5 mdeg

Chiroptical sensing results: 33.3 mM, 65.5:34.5 *er*

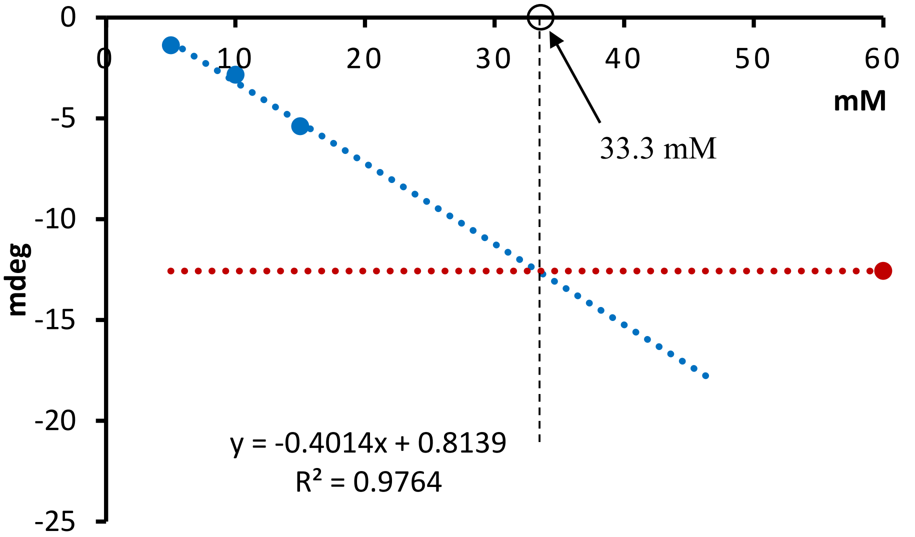

Figure S64. Variation of the 3rd measurement

The ICD (mdeg) value of the 3rd measurement was increased by +0.5 mdeg

Chiroptical sensing results: 36.7 mM, 67.0:33.0 *er*

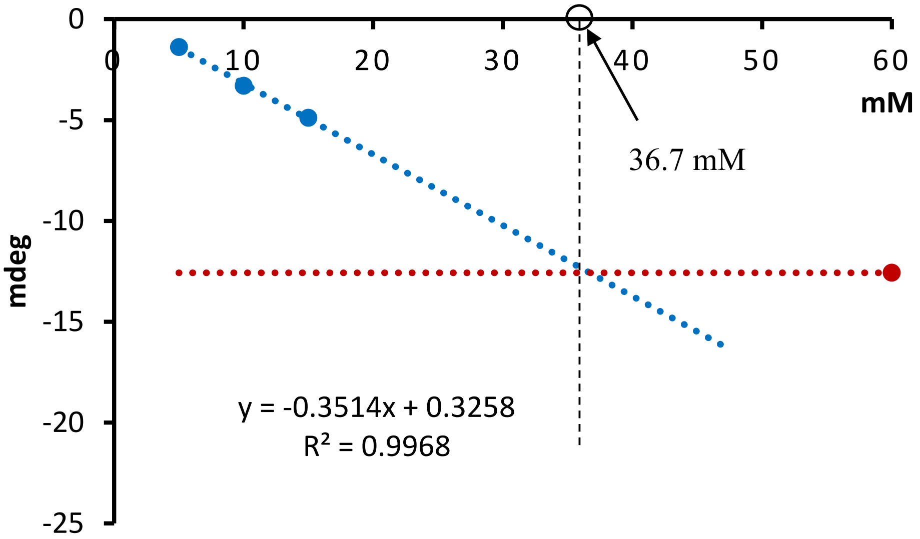

Figure S65. Variation of the 4th measurement

The ICD (mdeg) value of the 4th measurement was decreased by -0.5 mdeg

Chiroptical sensing results: 34.2 mM, 65.5:34.5 *er*

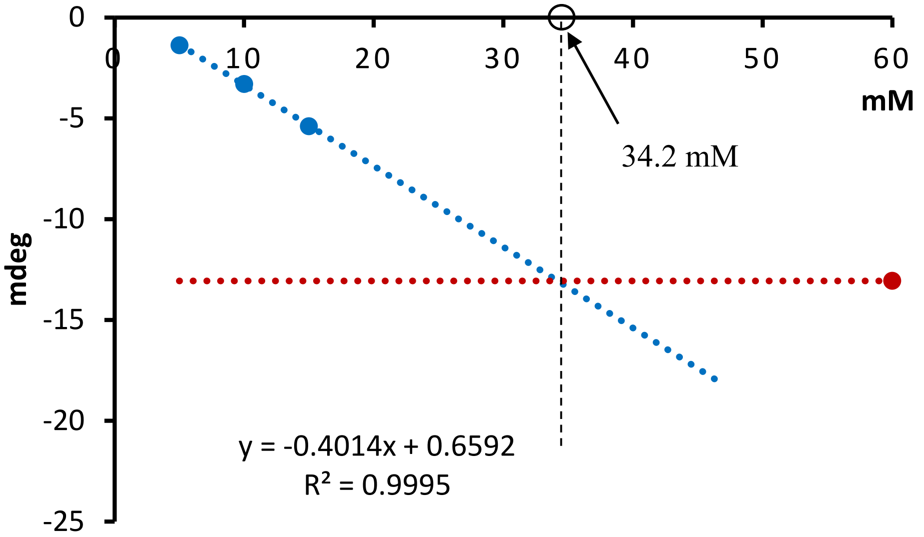

Supplement: Supplementary file 1 — jo4c02055_si_001.pdf [file jo4c02055_si_001.pdf]
